# Crucial but Neglected: Limited Availability of Animal Welfare Courses in Education of Wildlife Researchers

**DOI:** 10.3390/ani13182907

**Published:** 2023-09-13

**Authors:** Miriam A. Zemanova

**Affiliations:** 1 Environmental Sciences and Humanities Institute, University of Fribourg, Chemin du Musée 4, 1700 Fribourg, Switzerland; miriam.andela.zemanova@gmail.com; 2 Animalfree Research, Postgasse 15, 3011 Bern, Switzerland; 3 Oxford Centre for Animal Ethics, 91 Iffley Road, Oxford OX4 1EG, UK

**Keywords:** 3Rs principles, animal welfare, animal behaviour, animal physiology, animal health, education, wildlife welfare

## Abstract

**Simple Summary:**

Safeguarding animal welfare in research is crucial for ethical and legislative compliance as well as the integrity of scientific data. It is, therefore, essential that researchers working with animals across all fields of life sciences have an understanding of how to assess animal welfare, including their behaviour, health, and physiology. This study looked into the education of ecologists, wildlife biologists, and conservation managers in Europe, Canada, the USA, Australia, and New Zealand and found that very few universities offered specific courses on animal welfare, and these courses were often optional rather than required. These results highlight the need for universities to provide more formal and mandatory education on animal welfare to better prepare future researchers studying and managing wildlife. By improving education in this area, we can ensure that researchers have the necessary knowledge and skills to work with wildlife in a responsible and compassionate way.

**Abstract:**

Animal welfare is a subject of increasing scientific and ethical concern in today’s society, crucial for the well-being of animals used in research and the integrity of scientific data. Equipping researchers in the life science disciplines with a science-based knowledge of animal welfare, behaviour, physiology, and health is, therefore, essential. Nevertheless, previous studies evaluating animal welfare education focused on veterinary, laboratory, or farm animal science. Consequently, the aim of this study was, for the very first time, to map the prevalence of animal welfare courses in the university education of ecologists, wildlife biologists, and conservation managers in Europe, Canada, the USA, Australia, and New Zealand. A comprehensive assessment of 1548 universities was conducted, resulting in the identification of 596 relevant programs at the bachelor’s and master’s levels. Analysis of the curricula revealed that only 1% of the programs offered a formal course on animal welfare, while 65% provided courses on animal behaviour, 59% on animal physiology, and 34% on animal health. However, the majority of these courses were listed as electives rather than mandatory components of the programs. These results underscore the need for universities to incorporate more formal and obligatory education in animal welfare in order to better prepare future ecologists, wildlife biologists, and conservation managers for the challenges of working with wildlife.

## 1. Introduction

Understanding and protecting the many different species that live on Earth depend heavily on research in ecology, wildlife biology, and species conservation. This research offers insightful information on population dynamics, animal behaviour, ecological processes, and the effects of human activity on wildlife [1,2,3,4,5]. However, many conventional conservation research and management techniques might inadvertently inflict harm on the very animals they aim to protect. For instance, wildlife research can include chasing, darting, and capture methods as well as mutilations (e.g., through toe- or fin-clipping) for identification and tissue sampling, tagging, marking, and hot- or freeze-branding, considered to be necessary to gain more knowledge about animals’ biology or behaviour [6]. Additionally, animal welfare issues arise in species management, either with the removal of unwanted species with lethal means or with translocations and reintroductions, which include harm caused by capture, group separation, or stress of the source animals [7,8,9]. Furthermore, the welfare of individuals within socially complex species and genetically depauperate populations can be profoundly impacted, underscoring the importance of post-release welfare monitoring [10].

While wildlife research and conservation efforts are critically important, we should, nevertheless, be cognisant of potential animal welfare implications that might arise within these endeavours and attempt to mitigate them [11]. Inadequate knowledge and training in animal welfare can result in unintended harm to the animals involved in research and management, not only compromising the well-being of animals but also potentially undermining the validity of research outcomes [6]. Furthermore, legislations in many countries worldwide make it a prerequisite for research on vertebrate and some invertebrate animals to implement the 3Rs principles [12]: replacing the use of animals in experiments with other approaches whenever possible, reducing the number of animals used whilst ensuring the statistical power, and refining the experiments to minimise pain, suffering, distress, or lasting harm. The implementation of the 3Rs principles is stipulated within the EU by the Directive 2010/63/EU, in the USA in the USDA Animal Welfare Regulations [13], in Australia in a national standard unifying the different States’ Regulations [14], and in New Zealand in the Animal Welfare Act 1999. Therefore, anyone conducting research on animals has the ethical, scientific, as well as legal obligation to minimise animal welfare impacts. A crucial prerequisite for upholding good animal welfare standards is, however, a thorough understanding of animal behaviour and knowledge of physiological stress and health concepts [15].

The ethical imperative to address these concerns through education is increasingly recognised. While significant attention has been directed towards advocating for the integration of animal welfare courses within veterinary and animal science curricula [16,17,18,19,20], the training of ecologists, wildlife biologists, and conservation managers has been neglected. For instance, a recent study [21] reported that the availability of animal welfare courses for ecologists might be low, with only 38% of respondents in a survey stating that the topic of animal welfare was covered in their training and education. Although veterinarians could play a crucial role in ensuring the welfare of animals, they may not always be integral members of ecological research or conservation management teams. In 2013, Cattet [22] assessed 11 representative wildlife journals and found that only 26 out of 100 articles with an animal welfare focus included a co-author with a veterinary degree. Therefore, nurturing a foundational understanding of animal welfare principles becomes indispensable for ecologists and wildlife or conservation biologists who regularly interact with wildlife.

Despite this pressing need, a comprehensive evaluation of the prevalence of animal welfare courses in ecology, wildlife biology, and species conservation-related programs has not been attempted before. For that reason, the aim of this study was, for the first time, to explore the extent to which universities in Europe, Australia, New Zealand, Canada, and the USA have incorporated courses related to animal welfare, behaviour, physiology, and health into the curricula of programs training future ecologists, wildlife biologists, and conservation managers.

## 2. Materials and Methods

To assess the prevalence of courses relevant to animal welfare, I surveyed bachelor’s or master’s-level programs offered at universities in Europe, North America, and Oceania. This focus on developed countries with a similar level of animal welfare protection enabled a global comparison. The assessment was conducted between April and June 2023. Universities were identified through the university lists outlined on Wikipedia [23]. Universities with a clear medical, veterinary, law, business, or other unrelated focus—based on their name—were excluded from the evaluation. If the university offered a program likely to train ecologists, wildlife biologists, or conservation managers, for example, a bachelor’s or master’s degree in biodiversity, conservation biology, ecology, environmental biology, marine biology, organismal biology, wildlife biology, wildlife conservation, wildlife management, or zoology, the curriculum of this program was assessed for the presence of courses on animal welfare, animal behaviour, animal physiology, or animal health [24]. If the option to choose elective subjects was listed but without specification, the university’s course catalogue was examined to search for relevant elective subjects. The search was performed through a combination of reading through the catalogue and using a search function in order to minimise the chance of reporting false negatives. For classification, I followed the methodology described in Shivley et al. [25]: for a course to be categorised as an animal welfare course, its title needed to contain the term welfare or well-being. Courses in the animal behaviour area had to have the term behaviour, behavioural, or ethology in their title. For courses categorised as animal physiology, the term physiology, stress, or endocrinology had to be included in the title. Lastly, for courses in the animal health category, the term health, disease, or parasitology had to be present in the title. Descriptive statistics were implemented to summarise the results [25]. Fisher’s exact test was used to assess any potential influence of the region and education level (bachelor’s vs. master’s) on the prevalence of courses. This type of test was chosen to accommodate for low expected frequencies. The significance for all levels was set at *p* < 0.05, and *p* values were corrected for multiple testing using the Benjamini–Hochberg FDR method [26]. All statistical analyses were conducted in R 4.1.3 [27] integrated in RStudio 2022.02.1 [28].

## 3. Results

**Figure 1:**
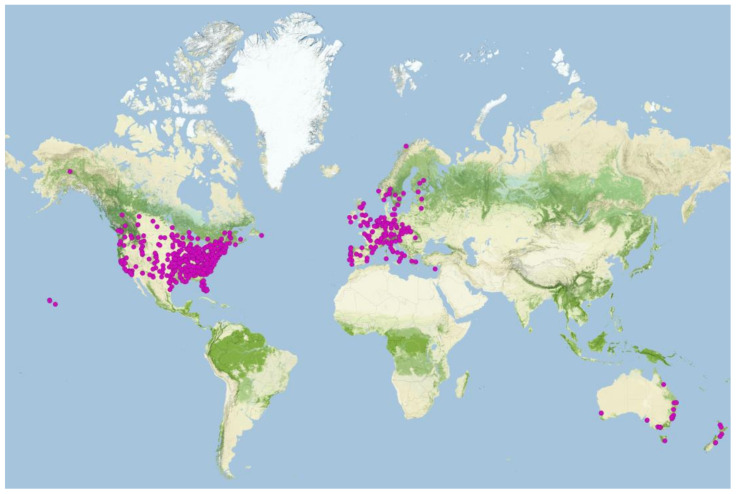
The purple dots indicate the location of the universities offering a bachelor’s or master’s level program in biodiversity, conservation biology, ecology, environmental biology, marine biology, organismal biology, wildlife biology, wildlife conservation, wildlife management, or zoology that were assessed for the presence of animal welfare-related courses (*N* = 596; see Table S1 for more details).

**Figure 2:**
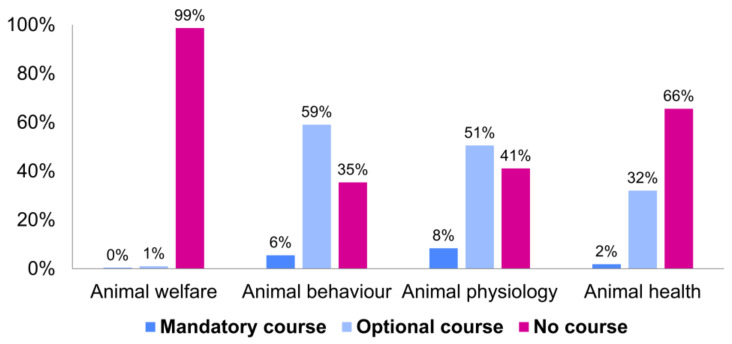
The proportion of the 596 programs that offer a mandatory, optional, or no course on animal welfare, animal behaviour, animal physiology, or animal health (see Table S1 for more details).

In total, 1548 universities were assessed across 33 countries (Table 1 and Tables S1 and S2). Excluded were universities that offered programs in general biology without a relevant specialisation or no wildlife-related programs (*N* = 802), environmental science programs focused entirely on abiotic aspects of the environment (*N* = 58), or programs without a curriculum available online (*N* = 92). As a curriculum was considered a list of subjects outlined on the program’s website. In total, 596 universities offered programs related to wildlife research and had their curricula accessible (Table 1 and Table S1; Figure 1). Only 8 of the 596 programs provided a formal course on animal welfare—2 of them as a mandatory course, 6 of them as an elective course (Table 2; Figure 2). A course on animal behaviour was offered at 385 programs, as a compulsory at 33 of them, and as an optional course at 352 (Table 2; Figure 2). An animal physiology course was listed in the curriculum of 351 programs: in 50 as a mandatory and in 301 as an elective course (Table 2; Figure 2). Animal health courses were included in 205 programs, out of which the courses in 11 programs were mandatory, and the courses in 194 programs were optional (Table 2; Figure 2). There was a statistically significant difference among regions, with all types of courses being most prevalent in North America (*p* < 0.001). Furthermore, the courses were more likely to be present at the bachelor’s level than at the master’s level: animal welfare courses were 4.23 times (*p* = 0.05), animal behaviour courses were 0.3 times (*p* < 0.001), animal physiology courses were 0.173 times (*p* < 0.001), and animal physiology courses were 0.432 times more likely to be present (*p* < 0.001).

## 4. Discussion

### 4.1. Prevalence of Animal Welfare Courses

This survey across 33 countries revealed that the availability of courses in animal welfare, animal behaviour, animal physiology, and animal health in university programs educating aspiring ecologists, wildlife biologists, and conservation managers is limited (Table 2; Figure 2). These findings are congruent with the results of a previous study reporting that animal welfare courses are not prevalent in the education of ecologists but would be appreciated [21]. The results show that bachelor’s level programs were more likely to incorporate animal welfare-related courses compared to master’s degrees. This might be a reflection of the more focused nature of master’s level programs, which often prioritise advanced topics relevant to a student’s chosen specialisation. It is nevertheless crucial that animal welfare education is included in both bachelor’s and master’s programs to ensure that all future ecologists, wildlife biologists, and conservation managers possess a well-rounded understanding of the subject.

### 4.2. Importance of Animal Welfare Education

There are several reasons why animal welfare courses should be included in education across all life science disciplines. First, animal welfare is an important ethical issue that is relevant to all fields that involve animal use, such as veterinary medicine, animal science, and wildlife biology [16]. The relevance for ecologists, wildlife biologists, and conservation managers stems from the potential animal welfare impact of commonly used research and management methods. For instance, the mere act of capture can induce substantial stress for free-living animals not accustomed to human contact [29], potentially leading to capture myopathy—a stress-associated metabolic disorder [9,30]. Also, blood sampling, toe-clipping, and attachment of radio transmitters have been reported to increase mortality, induce inflammation, or disrupt normal behaviour [31,32,33]. Apart from animal welfare considerations, robust scientific practices dictate that animals involved in research should remain unaffected by harm in terms of their physical, physiological, and behavioural well-being [34]. Any deviations from this norm can introduce alterations in an individual’s condition that may consequently influence the credibility, consistency, and replicability of experimental and observational outcomes [35]. Because of the above-mentioned animal welfare issues as well as animal protection regulations, many studies in ecology, wildlife biology, or species conservation projects are conducted by multidisciplinary teams. The participation of veterinarians, in particular, can greatly enhance the welfare of animals involved in research; however, this is not always the case [22]. The potential absence of experts in veterinary science highlights the importance of providing everyone involved in wildlife research and management with the knowledge and tools to mitigate potential risks to the animals.

Second, students who have a clear understanding of animal welfare issues early in their education are better equipped to make informed ethical decisions in their future careers [36,37]. The education should include not only an introduction to animal welfare and the most important concepts, such as Five Freedoms and Five Domains [38,39], but also the application of physiological, behavioural, and health indicators to situations that the students are most likely to encounter in their profession. For this reason, a collaboration between universities and zoos, wildlife parks, and nature reserves would be beneficial for the students to learn how to apply theoretical understanding of animal welfare in practice.

Third, animal welfare is of growing concern for the general public, and having a basic understanding of animal welfare issues can help scientists communicate their work to the public more effectively and transparently [11,20,40]. Some research and conservation management projects had to be halted in the past due to disagreement and outrage among the local communities [41,42].

Finally, incorporating animal welfare courses into life science curricula can help to promote a culture of respect and responsibility towards animals in the scientific community, leading to better animal care and more ethical research practices [43,44,45].

### 4.3. Recommendations and Future Directions

It is highly recommended that animal welfare be included as a mandatory subject in wildlife degree programs to fill the current vacuum. To guarantee that upcoming ecologists, wildlife biologists, and conservation managers receive thorough training in animal welfare, this integration should take place at both the undergraduate and graduate levels [16,46,47]. Educational institutions can emphasise the significance of animal welfare by giving it the same priority in the curriculum as other important topics in wildlife research and management, classifying it as a core subject [48]. Additionally, governmental and regulatory organisations, as well as wildlife research and conservation societies and scientific journals, ought to encourage and promote the inclusion of animal welfare education by acknowledging it as a crucial element of wildlife research and conservation [34].

It might be argued that students can be taught about animal welfare through informal discussions with lecturers and supervisors. While this could be a useful approach, it poses a couple of challenges: (1) the lecturer may not have received any animal welfare training either, and (2) the student might not understand the significance of the subject matter [49]. Consequently, some form of formal training in animal welfare is necessary and also provides transparency of what is being taught [25,49]. Furthermore, the importance of safeguarding animal welfare for ethical as well as scientific reasons should grant the subject a prominent place in the curriculum as a standalone course.

It is important to note that due to differences in attitudes towards animals, the course content related to animal welfare might vary between countries. As pointed out by Illmann et al. [50], countries with a rich tradition of animal welfare research may emphasise the fundamental scientific basis of animal welfare, including physiology and ethology, whereas, in countries that implement EU animal welfare policies but have less involvement in research, the focus may be more on practical aspects like legal issues and/or animal welfare assessment. Given the interdisciplinary nature of animal welfare, the incorporation of educators from various fields could provide valuable enrichment to the educational experience within each domain. The inclusion of guest speakers, particularly those engaged in roles spanning animal welfare, ecological research, and management, could further enhance the educational environment. The exact content of the courses should be investigated in future studies.

Implementing animal welfare education within ecology and wildlife conservation programs may face challenges and resistance. Scarce resources, curriculum restrictions, and opposition from established academic systems are some of the potential obstacles [51]. To overcome them, it is crucial to engage in proactive communication with academic institutions, faculty members, and other stakeholders. Support for integrating animal welfare education into wildlife degree programs can be gained by presenting arguments that are supported by data [21,52], highlighting successful examples from other institutions and programs [19,53,54], and emphasising the scientific and ethical necessity and the beneficial effects on research outcomes [6,10,22].

### 4.4. Study Limitations

In discussing the implications of the results, it is important to acknowledge the limitations inherent in the chosen methodology. The classification of courses based solely on their titles may not provide a comprehensive representation of the actual content and depth of animal welfare education. While every effort was made to categorise courses accurately, it is possible that some relevant courses were excluded. Furthermore, animal welfare courses that cater to ecologists might be provided by some veterinary faculties. Due to feasibility considerations, this study did not account for these nuances, and a more comprehensive search delving deeper into the actual content and pedagogy might provide more accurate estimates.

Another limitation is the focus on developed, higher-income countries, which prevents the generalisability of the results at a global scale. The findings of this study may not be directly applicable to developing countries, in which the educational system, resources, and priorities might be significantly different. The insights and recommendations mentioned could nevertheless provide some guidance for universities across the globe that aspire to improve animal welfare education.

## 5. Conclusions

Animal welfare should be an integral part of the education of all researchers who work with animals. However, this first overview of wildlife-related programs across several continents revealed that this subject remains neglected. This is a serious problem that needs to be addressed. Academic institutions and governing bodies should support the inclusion of animal welfare education in order to help create a new generation of ecologists, wildlife biologists, and conservation managers with the knowledge, abilities, and moral compass necessary to conduct research that upholds both animal welfare and the long-term conservation of wildlife populations.

## Figures and Tables

**Table 1:** The number of universities assessed in Europe, Canada, USA, Australia, and New Zealand, and the number of relevant programs identified and evaluated (see Tables S1 and S2 for more details).

Region	Country	Universities Assessed	Relevant Programs
Europe	Austria	5	5
Europe	Belgium	5	3
Europe	Bulgaria	4	0
Europe	Croatia	5	1
Europe	Cyprus	4	1
Europe	Czech Republic	8	6
Europe	Denmark	4	0
Europe	Estonia	3	1
Europe	Finland	5	3
Europe	France	32	14
Europe	Germany	47	22
Europe	Greece	4	2
Europe	Hungary	6	0
Europe	Ireland	6	3
Europe	Italy	33	15
Europe	Latvia	2	1
Europe	Lithuania	3	0
Europe	Malta	1	0
Europe	Netherlands	7	7
Europe	Norway	12	7
Europe	Poland	19	2
Europe	Portugal	10	7
Europe	Romania	6	1
Europe	Slovakia	6	4
Europe	Slovenia	1	1
Europe	Spain	31	9
Europe	Sweden	11	5
Europe	Switzerland	12	4
Europe	UK	14	14
North America	Canada	72	37
North America	USA	1127	391
Oceania	Australia	35	23
Oceania	New Zealand	8	7

**Table 2:** The number of mandatory, optional, and no courses on animal welfare, animal behaviour, animal physiology, and animal health categorised by region and level of the program (BSc/BA: bachelor’s degree; MSc/MA: master’s degree; see Table S1 for more details).

	Europe	North America	Oceania	BSc/BA	MSc/MA
**Animal welfare**					
Mandatory	1	0	1	1	1
Optional	4	1	1	3	3
None	133	427	28	476	112
**Animal behaviour**					
Mandatory	16	13	4	21	12
Optional	48	291	13	316	36
None	74	124	13	143	68
**Animal physiology**					
Mandatory	15	31	4	39	11
Optional	26	268	7	282	19
None	97	129	19	159	86
**Animal health**					
Mandatory	6	3	2	5	6
Optional	28	165	1	176	18
None	104	260	27	299	92

## Data Availability

The data presented in this study are available in the Supplementary Materials and upon request from the corresponding author.

## Supplementary Materials

The following supporting information can be downloaded at https://www.mdpi.com/article/10.3390/ani13182907/s1. Table S1: List of assessed programs at universities in Europe, Canada, USA, Australia, and New Zealand; Table S2: List of universities evaluated but not included.
