# Supplementary material for: Crucial but Neglected: Limited Availability of Animal Welfare Courses in Education of Wildlife Researchers
Source: Animals (Basel). 2023 Sep 13;13(18):2907. doi: 10.3390/ani13182907 (PMC10525554; doi:10.3390/ani13182907)
Supplement: Supplementary file 1 [file animals-13-02907-s001.zip › Supplementary Table S1.pdf]

**Table S1:** List of assessed programs at universities in Europe, Canada, USA, Australia, and New Zealand.

| Region | Country | University | Program name | Level |
|---|---|---|---|---|
| Europe | Austria | University of Graz | Ecology and Evolutionary Biology | MSc |
| Europe | Austria | University of Innsbruck | Ecology and Biodiversity | MSc |
| Europe | Austria | University of Natural Resources and Life Sciences | Wildlife Ecology and Wildlife Management | MSc |
| Europe | Austria | University of Salzburg | Ecology and Evolution | MSc |
| Europe | Austria | University of Vienna | Ecology and Ecosystems | MSc |
| Europe | Belgium | Catholic University of Louvain | Biology of Organisms and Ecology | MSc |
| Europe | Belgium | Free University of Brussels | Biology of Organisms and Ecology | MSc |
| Europe | Belgium | University of Mons | Biology of Organisms and Ecology | MSc |
| Europe | Croatia | University of Zagreb | Ecology and Nature Protection | BSc |
| Europe | Cyprus | University of Cyprus | Biodiversity and Ecology | MSc |
| Europe | Czech Republic | Charles University | Ecology and Evolutionary Biology | BSc |
| Europe | Czech Republic | Czech University of Life Sciences Prague | Applied Ecology | BSc |
| Europe | Czech Republic | Jan Evangelista Purkyně University in Ústí nad Labem | Applied Biology and Ecology | BSc |
| Europe | Czech Republic | Masaryk University | Ecology and Evolutionary Biology | BSc |
| Europe | Czech Republic | Palacký University Olomouc | Ecology and Environmental Protection | BSc |
| Europe | Czech Republic | University of Ostrava | Systematic Biology and Ecology | BSc |
| Europe | Estonia | University of Tartu | Biology and Biodiversity Protection | BSc |
| Europe | Finland | University of Eastern Finland | Biology of Environmental Change | MSc |
| Europe | Finland | University of Helsinki | Ecology and Evolutionary Biology | MSc |
| Europe | Finland | University of Jyväskylä | Biological and Environmental Science, specialization in Ecology and Evolutionary Biology | MSc |
| Europe | France | Claude Bernard University Lyon 1 | Biodiversity, Ecology and Evolution | MSc |
| Europe | France | Grenoble Alpes University | Biodiversity, Ecology, Evolution | MSc |
| Europe | France | Jean Monnet University | Ecology and Ethology | MSc |
| Europe | France | Paris-Saclay University | Biodiversity, Ecology and Evolution | MSc |
| Europe | France | Sorbonne University | Biodiversity, Ecology and Evolution | MSc |
| Europe | France | Toulouse III - Paul Sabatier University | Ecology and Evolution | MSc |
| Europe | France | University of Angers | Biodiversity, Ecology and Evolution | MSc |
| Europe | France | University of Lille | Biodiversity, Ecology and Evolution | MSc |
| Europe | France | University of Limoges | Biology of Organisms, Populations and Ecosystems | BSc |
| Europe | France | University of Montpellier | Ecology and Biodiversity Management | MSc |
| Europe | France | University of Perpignan Via Domitia | Biodiversity, Ecology, Evolution | MSc |
| Europe | France | University of Poitiers | Biodiversity, Ecology and Evolution | MSc |
| Europe | France | University of Rennes 1 | Biodiversity, Ecology and Evolution | MSc |
| Europe | France | University of Tours | Evolutionary and Behavioural Ecology | MSc |
| Europe | Germany | Bielefeld University | Ecology and Environmental Change | MSc |
| Europe | Germany | Free University of Berlin | Biodiversity, Evolution and Ecology | MSc |
| Europe | Germany | Goethe University Frankfurt | Ecology and Evolution | MSc |
| Europe | Germany | Ludwig Maximilian University of Munich | Evolution, Ecology and Systematics | MSc |
| Europe | Germany | Martin Luther University of Halle-Wittenberg | Biodiversity Sciences | MSc |
| Europe | Germany | Ruhr University Bochum | Biodiversity | MSc |
| Europe | Germany | Technische Universität Darmstadt | Biology, specialization in Ecology | MSc |
| Europe | Germany | TU Dresden | Organismic and Molecular Biodiversity | MSc |
| Europe | Germany | University of Bayreuth | Biodiversity and Ecology | MSc |
| Europe | Germany | University of Bonn | Organismic Biology, Evolutionary Biology and Palaeobiology | MSc |
| Europe | Germany | University of Duisburg-Essen | Biodiversity | MSc |
| Europe | Germany | University of Freiburg | Biology, specialization in Ecology and Evolution | MSc |
| Europe | Germany | University of Göttingen | Biodiversity, Ecology and Evolution | MSc |
| Europe | Germany | University of Greifswald | Biodiversity, Ecology and Evolution | MSc |
| Europe | Germany | University of Jena | Evolution, Ecology and Systematics | MSc |
| Europe | Germany | University of Kassel | Biology, specialization in Biodiversity and Evolution | MSc |
| Europe | Germany | University of Konstanz | Biology, specialization in Ecology, Evolution and Behaviour | MSc |
| Europe | Germany | University of Marburg | Biodiversity and Nature Conservation | MSc |
| Europe | Germany | University of Oldenburg | Landscape Ecology | MSc |
| Europe | Germany | University of Potsdam | Ecology, Evolution and Conservation | MSc |
| Europe | Germany | University of Rostock | Integrative Zoology | MSc |
| Europe | Germany | University of Tübingen | Evolution and Ecology | MSc |
| Europe | Greece | National and Kapodistrian University of Athens | Ecology and Biodiversity Management | MSc |
| Europe | Greece | University of Patras | Applied Ecology | MSc |
| Europe | Ireland | NUI Galway | Biodiversity & Land-Use Planning | MSc |
| Europe | Ireland | Trinity College Dublin | Biodiversity and Conservation | MSc |
| Europe | Ireland | University College Cork | Ecology and Environmental Biology | BSc |
| Europe | Italy | Roma Tre University | Biodiversity and Environmental Protection | MSc |
| Europe | Italy | Tuscia University | Marine Biology and Ecology | MSc |
| Europe | Italy | University of Bari | Environmental Biology | MSc |
| Europe | Italy | University of Bologna | Biodiversity and Evolution | MSc |
| Europe | Italy | University of Cagliari | Marine Bio-Ecology | MSc |
| Europe | Italy | University of Calabria | Biodiversity and Natural Systems | MSc |
| Europe | Italy | University of Messina | Biology and Ecology of the Coastal Marine Environment | MSc |
| Europe | Italy | University of Milan | Ecosystem Analysis, Monitoring and Management | MSc |
| Europe | Italy | University of Molise | Biodiversity | MSc |
| Europe | Italy | University of Padua | Evolutionary Biology | MSc |
| Europe | Italy | University of Parma | Ecology and Ethology for Nature Conservation | MSc |
| Europe | Italy | University of Salento | Biodiversity and Ecosystem Sciences | MSc |
| Europe | Italy | University of Siena | Biodiversity, Conservation and Environmental Quality | MSc |
| Europe | Italy | University of Trieste | Ecology and Sustainability of Global Changes | MSc |
| Europe | Italy | University of Turin | Environmental Biology | MSc |
| Europe | Latvia | University of Daugavpils | Biology, specialization in Biodiversity Research | MSc |
| Europe | Netherlands | Leiden University | Biodiversity and Sustainability | MSc |
| Europe | Netherlands | Radboud University | Conservation and Restoration Ecology | MSc |
| Europe | Netherlands | University of Amsterdam | Biological Sciences, specialization in Ecology and Evolution | MSc |
| Europe | Netherlands | University of Groningen | Ecology and Evolution, specialization in Ecology and Conservation | MSc |
| Europe | Netherlands | Utrecht University | Environmental Biology, specialization in Behavioural Ecology | MSc |
| Europe | Netherlands | Vrije Universiteit Amsterdam | Ecology and Evolution | MSc |
| Europe | Netherlands | Wageningen University & Research | Biology, specialization in Ecology and Biodiversity | MSc |
| Europe | Norway | Inland Norway University of Applied Sciences | Applied Ecology | MSc |
| Europe | Norway | Norwegian University of Life Sciences | Ecology | MSc |
| Europe | Norway | University of Agder | Coastal Ecology | MSc |
| Europe | Norway | University of Bergen | Biodiversity, Evolution and Ecology | MSc |
| Europe | Norway | University of Oslo | Biodiversity and Systematics | MSc |
| Europe | Norway | University of South-Eastern Norway | Ecology and Environmental Management | MSc |
| Europe | Norway | University of Tromsø | Biology, specialization in Ecology and Sustainability | MSc |
| Europe | Poland | Jagiellonian University | Ecology and Evolution | MSc |
| Europe | Poland | Wroclaw University of Environmental and Life Sciences | Environmental Biology | MSc |
| Europe | Portugal | University of Algarve | Biodiversity, Fisheries and Marine Conservation | MSc |
| Europe | Portugal | University of Aveiro | Applied Ecology | MSc |
| Europe | Portugal | University of Coimbra | Ecology | MSc |
| Europe | Portugal | University of Évora | Conservation Biology | MSc |
| Europe | Portugal | University of Lisbon | Conservation Biology | MSc |
| Europe | Portugal | University of Minho | Biodiversity, Ecology and Global Change | MSc |
| Europe | Portugal | University of Porto | Biodiversity, Genetics and Evolution | MSc |
| Europe | Romania | Babeș-Bolyai University | Terrestrial and Aquatic Ecology | MSc |
| Europe | Slovakia | Comenius University | Ecology and Environmental Protection | BSc |
| Europe | Slovakia | Matej Bel University | Environmental Biology | BSc |
| Europe | Slovakia | Pavol Jozef Šafárik University | Zoology and Animal Physiology | MSc |
| Europe | Slovakia | University of Prešov | Ecology and Environmental Science | BSc |
| Europe | Slovenia | University of Primorska | Nature Protection | MSc |
| Europe | Spain | Autonomous University of Barcelona | Terrestrial Ecology and Biodiversity Management | MSc |
| Europe | Spain | Complutense University of Madrid | Conservation Biology | MSc |
| Europe | Spain | University of A Coruña | Terrestrial Biodiversity | MSc |
| Europe | Spain | University of Alicante | Conservation of Biodiversity and Restoration of the Marine and Terrestrial Environment | MSc |
| Europe | Spain | University of Barcelona | Ecology, Management and Restoration of the Natural Environment | MSc |
| Europe | Spain | University of Salamanca | Biology and Conservation of Biodiversity | MSc |
| Europe | Spain | University of Santiago de Compostela | Terrestrial Biodiversity | MSc |
| Europe | Spain | University of the Basque Country | Biodiversity, Functioning and Management of Ecosystems | MSc |
| Europe | Spain | University of Valencia | Biodiversity: Conservation and Evolution | MSc |

| | | | | |
|---|---|---|---|---|
| Europe | Sweden | Karlstad University | Ecology and Nature Conservation | MSc |
| Europe | Sweden | Linköping University | Applied Ethology and Animal Biology | MSc |
| Europe | Sweden | Linnaeus University | Evolutionary Ecology | MSc |
| Europe | Sweden | Lund University | Animal Ecology | MSc |
| Europe | Sweden | Swedish University of Agricultural Sciences | Conservation and Management of Fish and Wildlife | MSc |
| Europe | Switzerland | ETH Zurich | Biology, specialization in Ecology and Evolution | MSc |
| Europe | Switzerland | University of Fribourg | Environmental Biology, specialization in Ecology and Evolution | MSc |
| Europe | Switzerland | University of Lausanne | Behaviour, Evolution and Conservation, specialization in Geosciences, Ecology and Environment | MSc |
| Europe | Switzerland | University of Zurich | Biology, specialization in Ecology | MSc |
| Europe | UK | Imperial College London | Ecology and Environmental Biology | BSc |
| Europe | UK | Lancaster University | Ecology and Conservation | BSc |
| Europe | UK | Royal Holloway University of London | Ecology and Conservation | BSc |
| Europe | UK | University of Aberdeen | Ecology | BSc |
| Europe | UK | University of Brighton | Ecology and Conservation | BSc |
| Europe | UK | University of East Anglia | Ecology and Conservation | BSc |
| Europe | UK | University of Edinburgh | Biological Sciences, specialization in Ecology | BSc |
| Europe | UK | University of Exeter | Conservation Biology and Ecology | BSc |
| Europe | UK | University of Gloucestershire | Ecology and Environmental Science | BSc |
| Europe | UK | University of Leeds | Ecology and Conservation Biology | BSc |
| Europe | UK | University of Reading | Ecology and Wildlife Conservation | BSc |
| Europe | UK | University of St Andrews | Ecology and Conservation | BSc |
| Europe | UK | University of Stirling | Ecology and Conservation | BSc |
| Europe | UK | University of York | Ecology | BSc |
| North America | Canada | Capilano University | Biology, specialization in Environmental Science | BSc |
| North America | Canada | Carleton University | Biology, specialization in Ecology, Evolution and Behaviour | BSc |
| North America | Canada | Concordia University | Ecology | BSc |
| North America | Canada | Lakehead University | Biology, specialization in Biodiversity and Conservation | BSc |
| North America | Canada | MacEwan University | Biology, specialization in Ecology and Diversity | BSc |
| North America | Canada | McGill University | Agricultural and Environmental Sciences, specialization in Wildlife Biology | BSc |
| North America | Canada | Memorial University of Newfoundland | Biology, specialization in Evolutionary Ecology | BSc |
| North America | Canada | Mount Royal University | Biology, specialization in Ecology and Evolution | BSc |
| North America | Canada | Nipissing University | Environmental Biology and Technology | BSc |
| North America | Canada | Queen's University at Kingston | Biology, specialization in Ecology and Evolutionary Biology | BSc |
| North America | Canada | Simon Fraser University | Biology, specialization in Ecology, Evolution, and Conservation | BSc |
| North America | Canada | Thompson Rivers University | Ecology and Environmental Biology | BSc |
| North America | Canada | Toronto Metropolitan University | Biology, specialization in Environmental Ecology | BSc |
| North America | Canada | Trent University | Biology, specialization in Conservation Biology | BSc |
| North America | Canada | Université de Montréal | Biology, specialization in Biodiversity, Ecology and Evolution | BSc |
| North America | Canada | Université de Sherbrooke | Ecology | BSc |
| North America | Canada | Université du Québec à Chicoutimi | Biology, specialization in Biodiversity and Ecosystems | BSc |
| North America | Canada | Université du Québec à Montréal | Biology, specialization in Ecology | BSc |
| North America | Canada | Université du Québec à Rimouski | Biology, specialization in Ecology | BSc |
| North America | Canada | Université du Québec à Trois-Rivières | Biological and Ecological Sciences | BSc |
| North America | Canada | Université Sainte-Anne | Biology, specialization in Environmental Biology | BSc |
| North America | Canada | University of Alberta | Environmental and Conservation Sciences, specialization in Conservation Biology | BSc |
| North America | Canada | University of British Columbia | Ecology, Evolution, and Conservation Biology | BSc |
| North America | Canada | University of Calgary | Biology, specialization in Biodiversity and Conservation | BSc |
| North America | Canada | University of Guelph | Wildlife Biology and Conservation | BSc |
| North America | Canada | University of Manitoba | Ecology and Environmental Biology | BSc |
| North America | Canada | University of Northern British Columbia | Conservation Science and Practice | BSc |
| North America | Canada | University of Ottawa | Biology, specialization in Ecology, Evolution, Behaviour | BSc |
| North America | Canada | University of Prince Edward Island | Wildlife Conservation | BSc |
| North America | Canada | University of Regina | Environmental Biology | BSc |
| North America | Canada | University of Saskatchewan | Environmental Biology | BSc |
| North America | Canada | University of the Fraser Valley | Biology, specialization in Ecology and Biology of Organisms | BSc |
| North America | Canada | University of Toronto | Ecology and Evolutionary Biology | BSc |
| North America | Canada | University of Western Ontario | Biology, specialization in Biodiversity and Conservation | BSc |
| North America | Canada | Vancouver Island University | Biology, specialization in Aquatic and Terrestrial Ecology | BSc |
| North America | Canada | Wilfrid Laurier University | Biology, specialization in Ecology, Evolution and Biodiversity | BSc |
| North America | Canada | York University | Environmental Biology | BSc |
| North America | USA | Abilene Christian University | Biology, specialization in Wildlife Biology | BSc |
| North America | USA | Adams State University | Biology, specialization in Wildlife | BSc |
| North America | USA | Alabama State University | Marine Biology | BSc |
| North America | USA | Alderson Broaddus University | Biology, specialization in Ecology | BSc |
| North America | USA | Alfred University | Biology, specialization in Ecology and Evolution | BSc |
| North America | USA | Allen University | Biology, specialization in Environmental Science | BSc |
| North America | USA | Anderson University (Indiana) | Biology, specialization in Ecology and Environmental Biology | BSc |
| North America | USA | Antioch University New England | Environmental Studies, specialization in Conservation Biology | MSc |
| North America | USA | Appalachian State University | Biology, specialization in Ecology, Evolution and Environmental Biology | BSc |
| North America | USA | Arcadia University | Biology, specialization in Conservation Ecology | BSc |
| North America | USA | Arizona State University | Biological Sciences, specialization in Conservation Biology and Ecology | BSc |
| North America | USA | Arkansas State University | Wildlife, Fisheries, and Conservation, specialization in Wildlife | BSc |
| North America | USA | Arkansas Tech University | Fisheries and Wildlife Sciences | BSc |
| North America | USA | Ashland University | Environmental Science, specialization in Biology | BSc |
| North America | USA | Athens State University | Biology, specialization in Ecology and Organismal Biology | BSc |
| North America | USA | Auburn University | Wildlife Ecology and Management | BSc |
| North America | USA | Augusta University | Ecology | BSc |
| North America | USA | Augustana University | Biology, specialization in Ecology and Environmental Science | BSc |
| North America | USA | Austin Peay State University | Biology, specialization in Ecology, Evolution, and Organismal Biology | BSc |
| North America | USA | Ave Maria University | Marine Biology | BSc |
| North America | USA | Baker University | Biology, specialization in Ecology | BSc |
| North America | USA | Ball State University | Biology, specialization in Wildlife Biology and Conservation | BSc |
| North America | USA | Barry University | Marine Biology | BSc |
| North America | USA | Belhaven University | Biology, specialization in Ecological Sciences | BSc |
| North America | USA | Bellarmine University | Biology, specialization in Organismal Biology | BSc |
| North America | USA | Belmont University | Biology, specialization in Ecology and Biodiversity | BSc |
| North America | USA | Bemidji State University | Wildlife Biology | BSc |
| North America | USA | Bethel University (Tennessee) | Biology, Field and Environment | BSc |
| North America | USA | Biola University | Biology, specialization in Environmental Science | BSc |
| North America | USA | Black Hills State University | Biology, specialization in Environmental Biology | BSc |
| North America | USA | Bloomsburg University of Pennsylvania | Ecology, Conservation and Field Biology | BSc |
| North America | USA | Bob Jones University | Forestry, specialization in Zoo and Wildlife Biology | BSc |
| North America | USA | Boise State University | Biology, specialization in Ecology, Evolution, and Behavior | BSc |
| North America | USA | Boston University | Biology, specialization in Ecology and Conservation Biology | BSc |
| North America | USA | Bowling Green State University | Biology, specialization in Ecology and Conservation Biology | BSc |
| North America | USA | Brigham Young University–Hawaii | Biology, specialization in Ecology, Evolution and Conservation Biology | BSc |
| North America | USA | Brigham Young University–Idaho | Fisheries, Range and Wildlife Management | BSc |
| North America | USA | Brown University | Biology, specialization in Ecology and Evolutionary Biology | BSc |
| North America | USA | Bryant University | Biology, specialization in Environmental Biology | BSc |
| North America | USA | California Polytechnic State University, San Luis Obispo | Environmental Management, specialization in Wildlife Biology | BSc |
| North America | USA | California State Polytechnic University, Humboldt | Wildlife, specialization in Conservation Biology and Applied Vertebrate Ecology | BSc |
| North America | USA | California State University San Marcos | Biology, specialization in Ecology | BSc |
| North America | USA | California State University, Fullerton | Biology, specialization in Ecology and Evolutionary Biology | BSc |
| North America | USA | California State University, Long Beach | Biology, specialization in Ecology, Evolution, and the Environment | BSc |
| North America | USA | California State University, Los Angeles | Biology, specialization in Organismal Biology | BSc |
| North America | USA | California State University, Monterey Bay | Biology, specialization in Ecology, Evolution, and Organismal Biology | BSc |
| North America | USA | California State University, Northridge | Biology, specialization in Ecology and Evolutionary Biology | BSc |
| North America | USA | California State University, Sacramento | Biology, specialization in Ecology, Evolution, and Conservation Biology | BSc |
| North America | USA | Calvin University | Environmental Health and Conservation, specialization in Ecology and Management | BSc |
| North America | USA | Cameron University | Biology, specialization in Organismal Biology | BSc |
| North America | USA | Capital University | Biology, specialization in Ecology and Environmental Science | BSc |
| North America | USA | Carlow University | Biology, specialization in Environmental Science and Management | BSc |
| North America | USA | Case Western Reserve University | Biology, specialization in Evolutionary Biology | BSc |
| North America | USA | Castleton University | Wildlife and Forest Conservation | BSc |
| North America | USA | Cedarville University | Biology, specialization in Environmental Biology | BSc |
| North America | USA | Central Connecticut State University | Biology, specialization in Ecology, Biodiversity, and Evolutionary Biology | BSc |
| North America | USA | Central Methodist University | Biology, specialization in Wildlife Ecology and Conservation | BSc |
| North America | USA | Central Michigan University | Biology, specialization in Ecology, Evolution, and Conservation | BSc |
| North America | USA | Central Washington University | Biology, specialization in Ecology and Evolutionary Biology | BSc |

| | | | | |
|---|---|---|---|---|
| North America | USA | Charleston Southern University | Forestry, specialization in Wildlife Biology | BSc |
| North America | USA | Christian Brothers University | Ecology | BSc |
| North America | USA | Christopher Newport University | Biology, specialization in Environmental Biology | BSc |
| North America | USA | Clemson University | Wildlife and Fisheries Biology | BSc |
| North America | USA | Cleveland State University | Environmental Science, specialization in Environmental Biology | BSc |
| North America | USA | Coastal Carolina University | Biology, specialization in Ecology, Evolution, and Conservation Biology | BSc |
| North America | USA | Coker University | Biology, specialization in Ecology | BSc |
| North America | USA | Colgate University | Environmental Biology | BSc |
| North America | USA | Colorado Mesa University | Biology, specialization in Ecology, Evolution, and Organismal Biology | BSc |
| North America | USA | Colorado State University | Fish, Wildlife, and Conservation Biology | BSc |
| North America | USA | Colorado State University Pueblo | Widlife and Natural Resources | BSc |
| North America | USA | Columbia University | Environmental Biology, specialization in Ecology and Evolution | BSc |
| North America | USA | Concord University | Biology, specialization in Field and Organismal Biology | BSc |
| North America | USA | Concordia University Texas | Environmental Science and Conservation | BSc |
| North America | USA | Concordia University Wisconsin | Environmental Science, specialization in Ecology | BSc |
| North America | USA | Cornell University | Biology, specialization in Ecology and Evolutionary Biology | BSc |
| North America | USA | Cornerstone University | Environmental Biology, specialization in Wildlife Biology | BSc |
| North America | USA | Creighton University | Environmental Science, specialization in Organismal and Population Ecology | BSc |
| North America | USA | Delaware State University | Natural Resources, specialization in Wildlife Management | BSc |
| North America | USA | Delaware Valley University | Biology, specialization in Zoology | BSc |
| North America | USA | Delta State University | Environmental Science, specialization in Wildlife Management | BSc |
| North America | USA | Denison University | Environmental Studies, specialization in Ecosystem Conservation | BSc |
| North America | USA | DePaul University | Biological Sciences, specialization in Ecology and Evolution | BSc |
| North America | USA | DePauw University | Biology, specialization in Environmental Biology | BSc |
| North America | USA | Dickinson State University | Biology, specialization in Organismal Biology | BSc |
| North America | USA | Dominican University of California | Biology, specialization in Ecology and Environmental Science | BSc |
| North America | USA | Drake University | Environmental Science, specialization in Zoo and Conservation Science | BSc |
| North America | USA | Drexel University | Biology, specialization in Ecology, Evolution and Genomics | BSc |
| North America | USA | Drury University | Environmental Biology | BSc |
| North America | USA | Duke University | Marine Science and Conservation | BSc |
| North America | USA | Duquesne University | Biology, specialization in Evolution and Organismal Biology | BSc |
| North America | USA | East Carolina University | Biology, specialization in Ecology and Evolution | BSc |
| North America | USA | East Central University | Biology, specialization in Ecology | BSc |
| North America | USA | East Tennessee State University | Biology, specialization in Natural Resource Ecology | BSc |
| North America | USA | Eastern Illinois University | Environmental Biology | BSc |
| North America | USA | Eastern Kentucky University | Biology, specialization in Biodiversity and Conservation | BSc |
| North America | USA | Eastern Michigan University | Ecology, Evolution and Organismal Biology | MSc |
| North America | USA | Eastern New Mexico University | Wildlife and Fisheries Science | BSc |
| North America | USA | Eastern Oregon University | Biology, specialization in Ecological Biology | BSc |
| North America | USA | Eastern Washington University | Biology, specialization in Wildlife Biology | BSc |
| North America | USA | Edgewood College | Environmental Science, specialization in Biology | BSc |
| North America | USA | Emory University | Biology, specialization in Ecology and Evolutionary Biology | BSc |
| North America | USA | Emporia State University | Biology, specialization in Ecology and Biodiversity | BSc |
| North America | USA | Fairfield University | Biology, specialization in Ecology, Evolution and Biodiversity | BSc |
| North America | USA | Fairleigh Dickinson University | Marine Biology | BSc |
| North America | USA | Fitchburg State University | Biology, specialization in Environmental Biology | BSc |
| North America | USA | Florida International University | Marine Biology | BSc |
| North America | USA | Florida State University | Biology, specialization in Ecology, Evolutionary Biology and Environmental Science | BSc |
| North America | USA | Fort Hays State University | Biology, specialization in Natural Resources | BSc |
| North America | USA | Fort Valley State University | Biology, specialization in Fisheries Biology and Wildlife Conservation | BSc |
| North America | USA | Frostburg State University | Wildlife and Fisheries | BSc |
| North America | USA | George Mason University | Biology, specialization in Environmental and Conservation Biology | BSc |
| North America | USA | George Washington University | Biology, specialization in Ecology, Evolution and Environment | BSc |
| North America | USA | Georgetown University | Biology, specialization in Ecology, Evolution and Behavioral Biology | BSc |
| North America | USA | Georgia College & State University | Biology, specialization in Marine Science | BSc |
| North America | USA | Georgia Southern University | Biology, specialization in Evolution and Ecology | BSc |
| North America | USA | Georgia State University | Biology, specialization in Ecology, Evolution and Organismal Biology | BSc |
| North America | USA | Grand Valley State University | Wildlife Biology | BSc |
| North America | USA | Greenville University | Environmental Biology | BSc |
| North America | USA | Hamline University | Biology, specialization in Ecology and Evolutionary Biology | BSc |
| North America | USA | Hampton University | Marine and Environmental Science | BSc |
| North America | USA | Hannibal–LaGrange University | Conservation Biology | BSc |
| North America | USA | Hardin–Simmons University | Biology, specialization in Wildlife and Field Biology | BSc |
| North America | USA | Hawaii Pacific University | Biology, specialization in Ecology and Evolution | BSc |
| North America | USA | Hofstra University | Biology, specialization in Ecology and Evolution | BSc |
| North America | USA | Howard University | Biology, specialization in Ecology and Evolution | BSc |
| North America | USA | Idaho State University | Biology, specialization in Ecology and Conservation | BSc |
| North America | USA | Illinois State University | Biology, specialization in Conservation Biology | BSc |
| North America | USA | Illinois Wesleyan University | Environmental Studies, specialization in Ecology | BSc |
| North America | USA | Indiana University Bloomington | Animal Behavior | BSc |
| North America | USA | Indiana University Northwest | Biology, specialization in Ecology and Conservation Biology | BSc |
| North America | USA | Indiana University of Pennsylvania | Biology, specialization in Ecology, Conservation and Evolutionary Biology | BSc |
| North America | USA | Iona University | Biology, specialization in Ecology and Environmental Biology | BSc |
| North America | USA | Iowa State University | Biology, specialization in Biodiversity and Evolution | BSc |
| North America | USA | Jackson State University | Biology, specialization in Environmental Science | BSc |
| North America | USA | Jacksonville State University | Biology, specialization in Ecology and Environmental Science | BSc |
| North America | USA | James Madison University | Biology, specialization in Ecology and Environmental Biology | BSc |
| North America | USA | Kansas State University | Fisheries, Wildlife, and Conservation Biology | BSc |
| North America | USA | Kean University | Biology, specialization in Environmental Biology | BSc |
| North America | USA | Kent State University | Zoology | BSc |
| North America | USA | Kutztown University of Pennsylvania | Biology, specialization in Organismal Biology and Ecology | BSc |
| North America | USA | Lake Superior State University | Conservation Biology | BSc |
| North America | USA | Liberty University | Zoo and Wildlife Biology | BSc |
| North America | USA | Lincoln Memorial University | Conservation Biology | BSc |
| North America | USA | Lindenwood University | Ecology and Evolutionary Biology | BSc |
| North America | USA | Lindenwood University – Belleville | Biological Sciences, specialization in Ecology and Evolutionary Biology | BSc |
| North America | USA | Lock Haven University of Pennsylvania | Biology, specialization in Ecology and Environmental Biology | BSc |
| North America | USA | Longwood University | Environmental Science, specialization in Life Science | BSc |
| North America | USA | Louisiana State University of Alexandria | Biology, specialization in Organismal and Field Biology | BSc |
| North America | USA | Loyola University Chicago | Biology, specialization in Ecology | BSc |
| North America | USA | Malone University | Zoo and Wildlife Biology | BSc |
| North America | USA | Mansfield University of Pennsylvania | Biology, specialization in Environmental Biology | BSc |
| North America | USA | Mars Hill University | Ecology and Conservation Biology | BSc |
| North America | USA | Marshall University | Biology, specialization in Ecology, Evolution, and Organismal Biology | BSc |
| North America | USA | Mayville State University | Biology, specialization in Wildlife | BSc |
| North America | USA | Michigan State University | Fisheries and Wildlife, specialization in Wildlife Biology and Management | BSc |
| North America | USA | Michigan Technological University | Wildlife Ecology and Conservation | BSc |
| North America | USA | Middle Tennessee State University | Biology, specialization in Organismal Biology and Ecology | BSc |
| North America | USA | Midwestern State University | Biology, specialization in Organismal Biology | BSc |
| North America | USA | Millersville University of Pennsylvania | Environmental Biology | BSc |
| North America | USA | Minnesota State University Moorhead | Biology, specialization in Ecology and Evolutionary Biology | BSc |
| North America | USA | Minnesota State University, Mankato | Biology, specialization in Ecology | BSc |
| North America | USA | Mississippi State University | Forestry, specialization in Wildlife Management | BSc |
| North America | USA | Missouri Southern State University | Biology, specialization in Ecology and Conservation | BSc |
| North America | USA | Missouri State University | Wildlife Conservation and Management | BSc |
| North America | USA | Missouri University of Science and Technology | Biology, specialization in Ecology, Environment and Conservation | BSc |
| North America | USA | Missouri Western State University | Wildlife Conservation and Management | BSc |
| North America | USA | Montana State University | Conservation Biology and Ecology | BSc |
| North America | USA | Montclair State University | Biology, specialization in Ecology and Evolution | MSc |
| North America | USA | Mount Vernon Nazarene University | Biology, specialization in Environmental Biology | BSc |
| North America | USA | Murray State University | Wildlife and Conservation Biology, specialization in Wildlife Biology | BSc |
| North America | USA | New Mexico Highlands University | Biology, specialization in Wildlife Biology and Conservation | BSc |
| North America | USA | New Mexico State University | Conservation Ecology | BSc |
| North America | USA | New York University | Biology, specialization in Ecology | BSc |
| North America | USA | Nicholls State University | Biology, specialization in Marine Biology | BSc |
| North America | USA | North Carolina State University | Biology, specialization in Ecology, Evolution and Conservation Biology | BSc |
| North America | USA | North Dakota State University | Biology, specialization in Ecology and Conservation | BSc |
| North America | USA | North Greenville University | Biology, specialization in Animal and Environmental Biology | BSc |
| North America | USA | Northeastern State University | Biology, specialization in Fish and Wildlife | BSc |

| | | | | |
|---|---|---|---|---|
| North America | USA | Northeastern University | Marine Biology | BSc |
| North America | USA | Northern Michigan University | Biology, specialization in Ecology | BSc |
| North America | USA | Northern Vermont University | Biology, specialization as Field Naturalist | BSc |
| North America | USA | Northwest Missouri State University | Wildlife Ecology and Conservation | BSc |
| North America | USA | Northwestern Oklahoma State University | Biology, specialization in Natural History | BSc |
| North America | USA | Northwestern State University | Biology, specialization in Natural Science | BSc |
| North America | USA | Northwestern University | Biology, specialization in Ecology, Evolution, and Conservation Biology | BSc |
| North America | USA | Ohio Northern University | Environmental and Field Biology | BSc |
| North America | USA | Ohio State University | Evolution and Ecology | BSc |
| North America | USA | Ohio University | Wildlife and Conservation Biology | BSc |
| North America | USA | Ohio Wesleyan University | Zoology | BSc |
| North America | USA | Oklahoma Panhandle State University | Animal Science, specialization in Wildlife Management | BSc |
| North America | USA | Oklahoma State University–Stillwater | Natural Resource Ecology and Management, specialization in Wildlife Ecology and Management | BSc |
| North America | USA | Old Dominion University | Marine Biology | BSc |
| North America | USA | Oregon State University | Fisheries, Wildlife, and Conservation Sciences | BSc |
| North America | USA | Otterbein University | Zoo and Conservation Science | BSc |
| North America | USA | Palm Beach Atlantic University | Biology, specialization in Botany, Environmental Science, and Field Biology | BSc |
| North America | USA | Pennsylvania State University | Biology, specialization in Ecology | BSc |
| North America | USA | Pennsylvania Western University | Conservation Ecology | BSc |
| North America | USA | Piedmont University | Biology, specialization in Ecology and Evolutionary Biology | BSc |
| North America | USA | Pittsburg State University | Biology, specialization in Wildlife Ecology and Conservation | BSc |
| North America | USA | Plymouth State University | Environmental Biology | BSc |
| North America | USA | Princeton University | Ecology and Evolutionary Biology | BSc |
| North America | USA | Purdue University | Wildlife | BSc |
| North America | USA | Purdue University Fort Wayne | Biology, specialization in Ecology and Evolutionary Biology | BSc |
| North America | USA | Queens University of Charlotte | Conservation Biology | BSc |
| North America | USA | Radford University | Biology, specialization in Wildlife Biology | BSc |
| North America | USA | Rice University | Biosciences, specialization in Ecology and Evolutionary Biology | BSc |
| North America | USA | Rivier University | Biology, specialization in Environmental Science | BSc |
| North America | USA | Rockford University | Biology, specialization in Conservation and Wildlife | BSc |
| North America | USA | Rockhurst University | Biology, specialization in Organismal Biology | BSc |
| North America | USA | Roger Williams University | Biology, specialization in Evolution and Ecology | BSc |
| North America | USA | Rogers State University | Biology, specialization in Environmental Conservation | BSc |
| North America | USA | Rutgers University | Ecology, Evolution, and Natural Resources | BSc |
| North America | USA | Saint Francis University | Biology, specialization in Ecology and Environmental Biology | BSc |
| North America | USA | Saint Louis University | Biology, specialization in Ecology, Evolution and Conservation | BSc |
| North America | USA | Saint Mary's University of Minnesota | Environmental Biology and Conservation | BA |
| North America | USA | Salem State University | Biology, specialization in Environmental Biology | BSc |
| North America | USA | Salisbury University | Biology, specialization in Environmental Biology | BSc |
| North America | USA | Salve Regina University | Biology, specialization in Environmental Sciences | BSc |
| North America | USA | San Francisco State University | Biology, specialization in Ecology, Evolution, and Conservation Biology | BSc |
| North America | USA | San Jose State University | Biology, specialization in Ecology and Evolution | BSc |
| North America | USA | Sewanee: The University of the South | Biology, specialization in Ecology and Biodiversity | BSc |
| North America | USA | Shawnee State University | Biology, specialization in Organismal Biology | BSc |
| North America | USA | Shippensburg University of Pennsylvania | Biology, specialization in Ecology, Environmental and Conservation Concentration | BSc |
| North America | USA | Shorter University | Ecology and Field Biology | BSc |
| North America | USA | Sonoma State University | Biology, specialization in Ecology and Evolutionary Biology | BSc |
| North America | USA | South Dakota State University | Ecology and Environmental Science | BSc |
| North America | USA | Southeast Missouri State University | Biology, specialization in Wildlife and Conservation Biology | BSc |
| North America | USA | Southeastern Oklahoma State University | Fisheries and Wildlife | BSc |
| North America | USA | Southern Arkansas University | Biology, specialization in Wildlife and Conservation Biology | BSc |
| North America | USA | Southern Illinois University Carbondale | Biological Sciences, specialization in Ecology | BSc |
| North America | USA | Southern Illinois University Edwardsville | Biology, specialization in Ecology, Evolution and Conservation | BSc |
| North America | USA | Southwest Baptist University | Biology, specialization in Environmental and Field Biology | BSc |
| North America | USA | Southwestern Adventist University | Biology, specialization in Ecology and Conservation Biology | BSc |
| North America | USA | St. Cloud State University | Biology, specialization in Biodiversity, Ecology and Evolution | BSc |
| North America | USA | St. Edward's University | Environmental Biology and Climate Change | BSc |
| North America | USA | St. Lawrence University | Conservation Biology | BSc |
| North America | USA | Stanford University | Biology, specialization in Ecology and Evolution | BSc |
| North America | USA | State University of New York at Cobleskill | Wildlife Management | BT |
| North America | USA | State University of New York at New Paltz | Biology, specialization in Organismal Biology | BSc |
| North America | USA | State University of New York at Oswego | Zoology | BSc |
| North America | USA | State University of New York at Plattsburgh | Ecology | BSc |
| North America | USA | State University of New York at Purchase | Biology, specialization in Evolutionary and Behavioral Biology | BSc |
| North America | USA | State University of New York College of Environmental Science and Forestry | Wildlife Science | BSc |
| North America | USA | Stockton University | Environmental Science, specialization in Wildlife Management | BSc |
| North America | USA | Stony Brook University | Environmental Studies, specialization in Conservation Biology | BSc |
| North America | USA | Suffolk University | Biology, specialization in Marine Science | BSc |
| North America | USA | SUNY Brockport | Environmental Science and Ecology | BSc |
| North America | USA | Susquehanna University | Ecology | BSc |
| North America | USA | Tarleton State University | Wildlife, Sustainability, and Ecosystem Sciences | BSc |
| North America | USA | Temple University | Ecology, Evolution and Biodiversity | BSc |
| North America | USA | Tennessee Tech | Wildlife and Fisheries Science, specialization in Conservation Biology | BSc |
| North America | USA | Texas A&M University | Ecology and Conservation Biology | BSc |
| North America | USA | Texas A&M University–Central Texas | Biology, specialization in Ecology and Conservation Biology | BSc |
| North America | USA | Texas A&M University–Commerce | Wildlife and Conservation Science | BSc |
| North America | USA | Texas A&M University–Corpus Christi | Biology, specialization in Ecology | BSc |
| North America | USA | Texas A&M University–San Antonio | Biology, specialization in Ecology | BSc |
| North America | USA | Texas State University | Wildlife Biology | BSc |
| North America | USA | Texas Tech University | Zoology | BSc |
| North America | USA | The Master's University | Biology, specialization in Natural History and Environmental Biology | BSc |
| North America | USA | Thomas More University | Biology, specialization in Marine Biology | BSc |
| North America | USA | Towson University | Biology, specialization in Ecology, Evolution and Conservation | BSc |
| North America | USA | Transylvania University | Biology, specialization in Ecology, Evolution, and Behavior | BSc |
| North America | USA | Trinity University (Texas) | Biology, specialization in Ecology and Evolution | BSc |
| North America | USA | Tulane University | Ecology and Evolutionary Biology | BSc |
| North America | USA | Union University | Conservation Biology | BSc |
| North America | USA | University of Alabama | Marine Science | BSc |
| North America | USA | University of Alabama at Birmingham | Biology, specialization in Marine Science | BSc |
| North America | USA | University of Alabama in Huntsville | Biology, specialization in Ecology and Evolution | BSc |
| North America | USA | University of Alaska Fairbanks | Biology, specialization in Ecology and Evolutionary Biology | BSc |
| North America | USA | University of Arizona | Ecology and Evolutionary Biology | BSc |
| North America | USA | University of Arkansas at Little Rock | Biology, specialization in Ecology and Organismal Biology | BSc |
| North America | USA | University of Bridgeport | Biology, specialization in Ecology, Conservation, Environmental Biology | BSc |
| North America | USA | University of California, Davis | Evolution, Ecology and Biodiversity | BA |
| North America | USA | University of California, Irvine | Ecology and Evolutionary Biology | BSc |
| North America | USA | University of California, Los Angeles | Ecology, Behavior and Evolution | BSc |
| North America | USA | University of California, Merced | Biology, specialization in Ecology and Evolutionary Biology | BSc |
| North America | USA | University of California, San Diego | Ecology, Behavior and Evolution | BSc |
| North America | USA | University of California, Santa Barbara | Ecology, Evolution, and Marine Biology | BSc |
| North America | USA | University of Central Arkansas | Environmental Science, specialization in Biology | BSc |
| North America | USA | University of Central Florida | Biology, specialization in Ecology, Evolutionary and Conservation Biology | BSc |
| North America | USA | University of Central Missouri | Biology, specialization in Wildlife and Natural Resource Conservation | BSc |
| North America | USA | University of Chicago | Biological Sciences, specialization in Ecology and Evolution | BSc |
| North America | USA | University of Cincinnati | Biology, specialization in Ecology and Evolution | BSc |
| North America | USA | University of Colorado Boulder | Ecology and Evolutionary Biology | BA |
| North America | USA | University of Connecticut | Ecology and Evolutionary Biology | BSc |
| North America | USA | University of Dallas | Biology, specialization in Environmental Science | BSc |
| North America | USA | University of Dayton | Biology, specialization in Environmental Biology and Ecology | BSc |
| North America | USA | University of Denver | Ecology and Biodiversity | BSc |
| North America | USA | University of Florida | Wildlife Ecology and Conservation | BSc |
| North America | USA | University of Georgia | Ecology | BSc |
| North America | USA | University of Hawai'i at Hilo | Biology, specialization in Ecology, Evolution and Conservation Biology | BSc |
| North America | USA | University of Houston–Clear Lake | Environmental Science, specialization in Environmental Biology | BSc |
| North America | USA | University of Idaho | Conservation Biology | BSc |
| North America | USA | University of Illinois Chicago | Biology, specialization in Evolution, Ecology and Environmental Biology | BSc |
| North America | USA | University of Kansas | Ecology, Evolution, and Organismal Biology | BA |
| North America | USA | University of Kentucky | Ecology and Evolutionary Biology | BSc |
| North America | USA | University of Louisiana at Lafayette | Biology, specialization in Ecology, Evolution and Marine Biology | BSc |

| | | | | |
|---|---|---|---|---|
| North America | USA | University of Maine | Wildlife Ecology | BSc |
| North America | USA | University of Maine at Farmington | Biology, specialization in Forest Ecology and Conservation | BSc |
| North America | USA | University of Maine at Fort Kent | Biology, specialization in Ecology and Conservation | BSc |
| North America | USA | University of Maine at Presque Isle | Biology, specialization in Ecology, Evolution and Biodiversity Conservation | BSc |
| North America | USA | University of Mary | Biology, specialization in Wildlife and Conservation Biology | BSc |
| North America | USA | University of Mary Washington | Environmental Science, specialization in Natural Science | BSc |
| North America | USA | University of Maryland Eastern Shore | Environmental Science, specialization in Marine Science | BSc |
| North America | USA | University of Maryland, College Park | Biology, specialization in Ecology and Evolution | BSc |
| North America | USA | University of Massachusetts Dartmouth | Biology, specialization in Ecology and Evolution Biology | BSc |
| North America | USA | University of Massachusetts Lowell | Biology, specialization in Ecology, Evolution and Organismal Biology | BSc |
| North America | USA | University of Miami | Marine Biology and Ecology | BSc |
| North America | USA | University of Michigan–Flint | Wildlife Biology | BSc |
| North America | USA | University of Minnesota | Ecology, Evolution and Behavior | BSc |
| North America | USA | University of Minnesota Morris | Biology, specialization in Environmental Biology | BSc |
| North America | USA | University of Mississippi | Biology, specialization in Ecology and Evolutionary Biology | BA |
| North America | USA | University of Montana | Biology, specialization in Ecology and Organismal Biology | BSc |
| North America | USA | University of Montana Western | Ecology | BSc |
| North America | USA | University of Mount Olive | Biology, specialization in Ecology and Environmental Science | BSc |
| North America | USA | University of Nebraska at Kearney | Biology, specialization in Wildlife | BSc |
| North America | USA | University of Nebraska–Lincoln | Fisheries and Wildlife | BSc |
| North America | USA | University of Nevada, Las Vegas | Biology, specialization in Ecology and Evolutionary Biology | BSc |
| North America | USA | University of Nevada, Reno | Wildlife Ecology and Conservation | BSc |
| North America | USA | University of New England (USA) | Biology, specialization in Ecology and Evolutionary Biology | BSc |
| North America | USA | University of New Mexico | Biology, specialization in Ecology, Evolution, and Organismal Biology | BSc |
| North America | USA | University of North Alabama | Biology, specialization in Environmental Biology | BSc |
| North America | USA | University of North Carolina at Greensboro | Biology, specialization in Environmental Biology | BSc |
| North America | USA | University of North Carolina at Pembroke | Biology, specialization in Zoology | BSc |
| North America | USA | University of North Carolina Wilmington | Marine Biology | BSc |
| North America | USA | University of North Dakota | Biology, specialization in Fisheries and Wildlife Biology | BSc |
| North America | USA | University of North Florida | Biology, specialization in Ecology and Evolution Biology | BSc |
| North America | USA | University of North Texas | Ecology for Environmental Science | BSc |
| North America | USA | University of Northern Iowa | Biology, specialization in Ecology, Evolution, and Organismal Biology | BSc |
| North America | USA | University of Notre Dame | Biology, specialization in Ecology and Environment | BSc |
| North America | USA | University of Oregon | Biology, specialization in Ecology and Evolution | BSc |
| North America | USA | University of Pennsylvania | Biology, specialization in Ecology and Evolutionary Biology | BA |
| North America | USA | University of Pittsburgh | Ecology and Evolution | BSc |
| North America | USA | University of Providence | Biology, specialization in Wildlife Ecology | BSc |
| North America | USA | University of Rio Grande | Wildlife Conservation | BSc |
| North America | USA | University of Saint Francis (Indiana) | Environmental Science, concentration in Conservation Biology | BSc |
| North America | USA | University of San Diego | Biology, specialization in Ecology and Evolutionary Biology | BSc |
| North America | USA | University of South Carolina Aiken | Biology, specialization in Environmental Remediation and Restoration | BSc |
| North America | USA | University of South Dakota | Conservation and Biodiversity | BSc |
| North America | USA | University of South Florida | Biology, specialization in Ecology and Evolution | BSc |
| North America | USA | University of Southern California | Biological Sciences, specialization in Ecology, Evolution and Environment | BSc |
| North America | USA | University of Southern Mississippi | Biological Sciences, specialization in Conservation Biology | BSc |
| North America | USA | University of Tampa | Biology, specialization in Organismal and Evolutionary Biology | BSc |
| North America | USA | University of Tennessee | Biology, specialization in Ecology and Evolutionary Biology | BSc |
| North America | USA | University of Tennessee at Martin | Ecology and Environmental Biology | BSc |
| North America | USA | University of Texas at Austin | Biology, specialization in Ecology, Evolution, and Behavior | BSc |
| North America | USA | University of Texas at El Paso | Ecology and Evolutionary Biology | BSc |
| North America | USA | University of Texas at San Antonio | Biology, specialization in Ecology | BSc |
| North America | USA | University of Texas Permian Basin | Biology, specialization in Organismal Biology | BSc |
| North America | USA | University of Texas Rio Grande Valley | Environmental Science, specialization in Environmental Biology | BSc |
| North America | USA | University of the Ozarks | Biology, specialization in Ecology and Wildlife Biology | BSc |
| North America | USA | University of Toledo | Biology, specialization in Ecology and Organismal Biology | BSc |
| North America | USA | University of Utah | Biology, specialization in Ecology, Evolution and Environmental Biology | BSc |
| North America | USA | University of Vermont | Zoology | BSc |
| North America | USA | University of West Alabama | Conservation and Field Biology | BSc |
| North America | USA | University of Wisconsin | Wildlife Ecology and Management | BSc |
| North America | USA | University of Wisconsin–Eau Claire | Biology, specialization in Ecology and Environmental Biology | BSc |
| North America | USA | University of Wisconsin–Green Bay | Biology, specialization in Ecology and Conservation | BSc |
| North America | USA | University of Wisconsin–Madison | Wildlife Ecology | BSc |
| North America | USA | University of Wisconsin–Milwaukee | Biology, specialization in Ecology, Evolution and Behavior | BSc |
| North America | USA | University of Wisconsin–Oshkosh | Biology, specialization in Ecology and Organismal Biology | BSc |
| North America | USA | University of Wisconsin–Parkside | Environmental Science, specialization in Biology | BSc |
| North America | USA | University of Wisconsin–Platteville | Environmental Science and Conservation | BSc |
| North America | USA | University of Wisconsin–River Falls | Biology, specialization in Ecology and Organismal Biology | BSc |
| North America | USA | University of Wisconsin–Superior | Biology, specialization in Ecology, Aquatic Biology and Fishery | BSc |
| North America | USA | University of Wisconsin–Whitewater | Biology, specialization in Ecology, Evolution, and Behavior | BSc |
| North America | USA | University of Wyoming | Biology, specialization in Ecology and Evolution | BSc |
| North America | USA | Utah State University | Biology, specialization in Ecology and Evolutionary Biology | BSc |
| North America | USA | Vanderbilt University | Ecology, Evolution, and Organismal Biology | BSc |
| North America | USA | Vanguard University | Biology, specialization in Ecology | BSc |
| North America | USA | Virginia Tech | Wildlife Conservation | BSc |
| North America | USA | Washburn University | Environmental Biology | BSc |
| North America | USA | Washington State University | Biology, specialization in Ecology and Evolutionary Biology | BSc |
| North America | USA | Washington University in St. Louis | Environmental Biology | BSc |
| North America | USA | Weber State University | Zoology | BSc |
| North America | USA | Webster University | Biology, specialization in Biodiversity | BA |
| North America | USA | West Chester University | Biology, specialization in Ecology and Conservation | BSc |
| North America | USA | West Liberty University | Ecology, Evolution, and Organismal Biology | BSc |
| North America | USA | West Texas A&M University | Wildlife Biology | BSc |
| North America | USA | West Virginia University | Biology, specialization in Ecology and Environmental Biology | BSc |
| North America | USA | Western Connecticut State University | Biology, specialization in Ecological Sciences | BSc |
| North America | USA | Western Illinois University | Biology, specialization in Environmental Biology | BSc |
| North America | USA | Western New Mexico University | Zoology | BSc |
| North America | USA | Western Washington University | Biology, specialization in Ecology, Evolution, and Organismal Biology | BSc |
| North America | USA | Wichita State University | Biology, specialization in Ecological / Environmental / Organismal Emphasis | BSc |
| North America | USA | William Paterson University | Biology, specialization in Ecology | BSc |
| North America | USA | Wingate University | Environmental Biology | BSc |
| North America | USA | Winona State University | Biology, specialization in Ecology | BSc |
| North America | USA | Winthrop University | Biology, specialization in Conservation Biology | BSc |
| North America | USA | Wright State University | Biology, specialization in Ecology, Evolution, and Organismal Biology | BSc |
| North America | USA | Yale University | Ecology and Evolutionary Biology | BSc |
| Oceania | Australia | Federation University Australia | Environmental and Conservation Science | BSc |
| Oceania | Australia | Flinders University | Biodiversity and Conservation | BSc |
| Oceania | Australia | Griffith University | Environmental Science, specialization in Ecology and Conservation | BSc |
| Oceania | Australia | James Cook University | Zoology and Ecology | BSc |
| Oceania | Australia | La Trobe University | Wildlife and Conservation Biology | BSc |
| Oceania | Australia | Macquarie University | Biodiversity and Conservation | BSc |
| Oceania | Australia | Monash University | Ecology and Conservation Biology | BSc |
| Oceania | Australia | Murdoch University | Conservation and Wildlife Biology | BSc |
| Oceania | Australia | University of Adelaide | Marine and Wildlife Conservation | BSc |
| Oceania | Australia | University of Melbourne | Ecology and Evolutionary Biology | BSc |
| Oceania | Australia | University of New England (Australia) | Zoology | BSc |
| Oceania | Australia | University of New South Wales | Environmental Management, specialization in Ecology | BSc |
| Oceania | Australia | University of Newcastle | Environmental Science and Management, specialization in Ecosystems and Biodiversity | BSc |
| Oceania | Australia | University of Queensland | Ecology and Conservation Biology | BSc |
| Oceania | Australia | University of Southern Queensland | Environmental Science, specialization in Ecology and Conservation | BSc |
| Oceania | Australia | University of Sydney | Wildlife Conservation | BSc |
| Oceania | Australia | University of Tasmania | Ecology | BSc |
| Oceania | Australia | University of Technology Sydney | Environmental Biology | BSc |
| Oceania | Australia | University of the Sunshine Coast | Animal Ecology | BSc |
| Oceania | Australia | University of Western Australia | Conservation Biology | BSc |
| Oceania | Australia | University of Wollongong | Ecology and Conservation Biology | BSc |
| Oceania | Australia | Victoria University, Melbourne | Ecology and Environmental Management | BSc |
| Oceania | Australia | Western Sydney University | Zoology | BSc |
| Oceania | New Zealand | Lincoln University | Conservation and Ecology | BSc |
| Oceania | New Zealand | Massey University | Ecology and Conservation | BSc |

| | | | | |
|---|---|---|---|---|
| Oceania | New Zealand | University of Auckland | Ecology | MSc |
| Oceania | New Zealand | University of Canterbury | Biological Sciences, specialization in Ecology, Evolution and Behaviour | BSc |
| Oceania | New Zealand | University of Otago | Ecology | BSc |
| Oceania | New Zealand | University of Waikato | Ecology and Biodiversity | BSc |
| Oceania | New Zealand | Victoria University of Wellington | Conservation Biology | MSc |
