# Supplementary material for: Crucial but Neglected: Limited Availability of Animal Welfare Courses in Education of Wildlife Researchers
Source: Animals (Basel). 2023 Sep 13;13(18):2907. doi: 10.3390/ani13182907 (PMC10525554; doi:10.3390/ani13182907)
Supplement: Supplementary file 1 [file animals-13-02907-s001.zip › Supplementary Table S2.pdf]

Table S2: List of universities evaluated but not included due to absence of a relevant program, website unavailability, or inaccessibility of curricula.

Region	Country	University
Europe	Belgium	Ghent University
Europe	Belgium	University of Leuven
Europe	Bulgaria	New Bulgarian University
Europe	Bulgaria	Plovdiv University "Paisii Hilendarski"
Europe	Bulgaria	Sofia University
Europe	Bulgaria	University of Ruse
Europe	Croatia	Juraj Dobrila University of Pula
Europe	Croatia	University of Osijek
Europe	Croatia	University of Split
Europe	Croatia	University of Zadar
Europe	Cyprus	Cyprus International University
Europe	Cyprus	European University Cyprus
Europe	Cyprus	University of Cyprus
Europe	Czech Republic	University of Hradec Králové
Europe	Czech Republic	University of South Bohemia in České Budějovice
Europe	Denmark	Aarhus University
Europe	Denmark	Roskilde University
Europe	Denmark	University of Copenhagen
Europe	Denmark	University of Southern Denmark
Europe	Estonia	Estonian University of Life Sciences
Europe	Estonia	Tallinn University
Europe	Finland	University of Oulu
Europe	Finland	University of Turku
Europe	France	Aix-Marseille University
Europe	France	Avignon University
Europe	France	Le Havre Normandy University
Europe	France	Le Mans University
Europe	France	Paris-East Créteil University
Europe	France	Savoy Mont Blanc University
Europe	France	Southern Brittany University
Europe	France	Toulouse III - Paul Sabatier University
Europe	France	University of Bordeaux
Europe	France	University of Burgundy
Europe	France	University of Caen Normandy
Europe	France	University of Évry Val d'Essonne
Europe	France	University of Nantes
Europe	France	University of Picardy Jules Verne
Europe	France	University of Reims Champagne-Ardenne
Europe	France	University of Strasbourg
Europe	France	University of Tours
Europe	France	University of Versailles Saint-Quentin-en-Yvelines
Europe	Germany	Chemnitz University of Technology
Europe	Germany	Heidelberg University
Europe	Germany	Heinrich Heine University Düsseldorf
Europe	Germany	Kiel University
Europe	Germany	Leibniz University Hannover
Europe	Germany	Leipzig University
Europe	Germany	Osnabrück University
Europe	Germany	Otto von Guericke University Magdeburg
Europe	Germany	RWTH Aachen University
Europe	Germany	Saarland University
Europe	Germany	Technical University of Munich
Europe	Germany	University of Cologne
Europe	Germany	University of Erlangen–Nuremberg
Europe	Germany	University of Giessen
Europe	Germany	University of Hamburg
Europe	Germany	University of Hildesheim
Europe	Germany	University of Hohenheim
Europe	Germany	University of Koblenz and Landau
Europe	Germany	University of Lübeck
Europe	Germany	University of Mainz
Europe	Germany	University of Münster
Europe	Germany	University of Regensburg
Europe	Germany	University of Ulm
Europe	Germany	University of Wuppertal
Europe	Germany	University of Würzburg
Europe	Greece	Aristotle University of Thessaloniki
Europe	Greece	University of Crete
Europe	Hungary	Eötvös Loránd University
Europe	Hungary	Eszterházy Károly Catholic University
Europe	Hungary	University of Debrecen

Europe	Hungary	University of Nyíregyháza
Europe	Hungary	University of Pécs
Europe	Hungary	University of Szeged
Europe	Ireland	Maynooth University
Europe	Ireland	University College Dublin
Europe	Ireland	University of Limerick
Europe	Italy	Marche Polytechnic University
Europe	Italy	Università degli Studi della Campania Luigi Vanvitelli
Europe	Italy	University of Basilicata
Europe	Italy	University of Camerino
Europe	Italy	University of Catania
Europe	Italy	University of Eastern Piedmont
Europe	Italy	University of Ferrara
Europe	Italy	University of Florence
Europe	Italy	University of Milano-Bicocca
Europe	Italy	University of Modena and Reggio Emilia
Europe	Italy	University of Palermo
Europe	Italy	University of Pavia
Europe	Italy	University of Perugia
Europe	Italy	University of Pisa
Europe	Italy	University of Rome Tor Vergata
Europe	Italy	University of Salerno
Europe	Italy	University of Sannio
Europe	Italy	University of Sassari
Europe	Latvia	University of Latvia
Europe	Lithuania	Klaipėda University
Europe	Lithuania	Vilnius University
Europe	Lithuania	Vytautas Magnus University
Europe	Malta	University of Malta
Europe	Norway	Norwegian University of Science and Technology
Europe	Norway	Østfold University College
Europe	Norway	Sámi University of Applied Sciences
Europe	Norway	University of Stavanger
Europe	Norway	Western Norway University of Applied Sciences
Europe	Poland	Adam Mickiewicz University in Poznań
Europe	Poland	Cardinal Stefan Wyszyński University in Warsaw
Europe	Poland	Jan Kochanowski University
Europe	Poland	Kazimierz Wielki University in Bydgoszcz
Europe	Poland	Maria Curie-Skłodowska University
Europe	Poland	Nicolaus Copernicus University in Toruń
Europe	Poland	University of Białystok
Europe	Poland	University of Gdańsk
Europe	Poland	University of Łódź
Europe	Poland	University of Opole
Europe	Poland	University of Rzeszów
Europe	Poland	University of Silesia in Katowice
Europe	Poland	University of Szczecin
Europe	Poland	University of Warmia and Mazury in Olsztyn
Europe	Poland	University of Warsaw
Europe	Poland	University of Wrocław
Europe	Poland	University of Zielona Góra
Europe	Portugal	NOVA University Lisbon
Europe	Portugal	University of Madeira
Europe	Portugal	University of Trás-os-Montes and Alto Douro
Europe	Romania	Alexandru Ioan Cuza University
Europe	Romania	Ovidius University
Europe	Romania	University of Bacău
Europe	Romania	University of Bucharest
Europe	Romania	West University of Timișoara
Europe	Slovakia	Constantine the Philosopher University in Nitra
Europe	Slovakia	University of Ss. Cyril and Methodius
Europe	Spain	University of Alcalá
Europe	Spain	University of Alicante
Europe	Spain	University of Almería
Europe	Spain	University of Burgos
Europe	Spain	University of Cádiz
Europe	Spain	University of Cantabria
Europe	Spain	University of Córdoba
Europe	Spain	University of Extremadura
Europe	Spain	University of Girona
Europe	Spain	University of Granada
Europe	Spain	University of Jaén
Europe	Spain	University of La Laguna
Europe	Spain	University of León
Europe	Spain	University of Málaga

Europe	Spain	University of Murcia
Europe	Spain	University of Navarra
Europe	Spain	University of Oviedo
Europe	Spain	University of Seville
Europe	Spain	University of the Balearic Islands
Europe	Spain	University of the Basque Country
Europe	Spain	University of Vigo
Europe	Spain	University of Zaragoza
Europe	Sweden	Mid Sweden University
Europe	Sweden	Örebro University
Europe	Sweden	Södertörn University
Europe	Sweden	Stockholm University
Europe	Sweden	University of Gothenburg
Europe	Sweden	Uppsala University
Europe	Switzerland	École Polytechnique Fédérale de Lausanne
Europe	Switzerland	Università della Svizzera italiana
Europe	Switzerland	University of Basel
Europe	Switzerland	University of Bern
Europe	Switzerland	University of Geneva
Europe	Switzerland	University of Lucerne
Europe	Switzerland	University of Neuchâtel
Europe	Switzerland	University of St. Gallen
North America	Canada	Acadia University
North America	Canada	Algoma University
North America	Canada	Athabasca University
North America	Canada	Bishop's University
North America	Canada	Brandon University
North America	Canada	Brock University
North America	Canada	Cape Breton University
North America	Canada	Dalhousie University
North America	Canada	Kwantlen Polytechnic University
North America	Canada	Laurentian University
North America	Canada	McMaster University
North America	Canada	Mount Allison University
North America	Canada	Mount Saint Vincent University
North America	Canada	Ontario Tech University
North America	Canada	Royal Roads University
North America	Canada	Saint Mary's University
North America	Canada	St. Francis Xavier University
North America	Canada	St. Thomas University
North America	Canada	Université de Hearst
North America	Canada	Université de l'Ontario français
North America	Canada	Université de Moncton
North America	Canada	Université de Saint-Boniface
North America	Canada	Université du Québec en Abitibi-Témiscamingue
North America	Canada	Université du Québec en Outaouais
North America	Canada	Université Laval
North America	Canada	University College of the North
North America	Canada	University of King's College
North America	Canada	University of Lethbridge
North America	Canada	University of New Brunswick
North America	Canada	University of Sudbury
North America	Canada	University of Victoria
North America	Canada	University of Waterloo
North America	Canada	University of Windsor
North America	Canada	University of Winnipeg
North America	Canada	Yukon University
North America	USA	A.T. Still University
North America	USA	Adelphi University
North America	USA	Adler University
North America	USA	Alabama A&M University
North America	USA	Alaska Pacific University
North America	USA	Albany State University
North America	USA	Albizu University
North America	USA	Alcorn State University
North America	USA	Alliance University (New York City)
North America	USA	Alliant International University
North America	USA	Alvernia University
North America	USA	Amberton University
North America	USA	American Heritage University of Southern California
North America	USA	American InterContinental University
North America	USA	American Jewish University
North America	USA	American National University
North America	USA	American University
North America	USA	Amridge University

North America	USA	Anaheim University
North America	USA	Anderson University (South Carolina)
North America	USA	Andrews University
North America	USA	Angelo State University
North America	USA	Antioch University
North America	USA	Antioch University Midwest
North America	USA	Apollos University
North America	USA	Argosy University
North America	USA	Arizona Christian University
North America	USA	Arkansas State University Mid-South
North America	USA	Arkansas State University Three Rivers
North America	USA	Arkansas State University-Beebe
North America	USA	Arkansas State University–Mountain Home
North America	USA	Arkansas State University-Newport
North America	USA	Arlington Baptist University
North America	USA	Asbury University
North America	USA	Assumption University (Worcester)
North America	USA	Atlantic International University
North America	USA	Atlantis University
North America	USA	Augsburg University
North America	USA	Aurora University
North America	USA	Averett University
North America	USA	Avila University
North America	USA	Azusa Pacific University
North America	USA	Bakke Graduate University
North America	USA	Baldwin Wallace University
North America	USA	Bay Path University
North America	USA	Baylor University
North America	USA	Beal University
North America	USA	Bellevue University
North America	USA	Benedictine University
North America	USA	Benjamin Franklin Cummings Institute of Technology
North America	USA	Bentley University
North America	USA	Bethany Global University
North America	USA	Bethel University (Indiana)
North America	USA	Bethel University (Minnesota)
North America	USA	Bethune–Cookman University
North America	USA	Binghamton University
North America	USA	Bluefield State University
North America	USA	Bluefield University
North America	USA	Bluffton University
North America	USA	Bowie State University
North America	USA	Bradley University
North America	USA	Brandeis University
North America	USA	Brenau University
North America	USA	Brescia University
North America	USA	Briar Cliff University
North America	USA	Bridgewater State University
North America	USA	Bryan University
North America	USA	Bucknell University
North America	USA	Buena Vista University
North America	USA	Buffalo State University
North America	USA	Bushnell University
North America	USA	Butler University
North America	USA	Cabrini University
North America	USA	Cairn University
North America	USA	Caldwell University
North America	USA	California Baptist University
North America	USA	California Coast University
North America	USA	California Lutheran University
North America	USA	California Miramar University
North America	USA	California South Bay University
North America	USA	California Southern University
North America	USA	California State Polytechnic University, Pomona
North America	USA	California State University Channel Islands
North America	USA	California State University, Bakersfield
North America	USA	California State University, Chico
North America	USA	California State University, Dominguez Hills
North America	USA	California State University, East Bay
North America	USA	California State University, Fresno
North America	USA	California State University, San Bernardino
North America	USA	California State University, Stanislaus
North America	USA	Campbell University
North America	USA	Campbellsville University
North America	USA	Capella University

North America	USA	Cardinal Stritch University
North America	USA	Carnegie Mellon University
North America	USA	Carolina University
North America	USA	Carroll University
North America	USA	Carson–Newman University
North America	USA	Catholic Distance University
North America	USA	Catholic University of America
North America	USA	Centenary College of Louisiana
North America	USA	Centenary University
North America	USA	Central State University
North America	USA	Chamberlain University
North America	USA	Chaminade University of Honolulu
North America	USA	Chapman University
North America	USA	Charles R. Drew University of Medicine and Science
North America	USA	Chatham University
North America	USA	Cheyney University of Pennsylvania
North America	USA	Chicago State University
North America	USA	Chowan University
North America	USA	Claflin University
North America	USA	Claremont Graduate University
North America	USA	Claremont Lincoln University
North America	USA	Clark Atlanta University
North America	USA	Clark University
North America	USA	Clarke University
North America	USA	Clarks Summit University
North America	USA	Clarkson University
North America	USA	Clayton State University
North America	USA	Cleary University
North America	USA	Cleveland University-Kansas City
North America	USA	College of Saint Benedict and Saint John's University
North America	USA	Colorado Christian University
North America	USA	Colorado Technical University
North America	USA	Columbia International University
North America	USA	Columbia Southern University
North America	USA	Columbus State University
North America	USA	Concordia University Ann Arbor
North America	USA	Concordia University Chicago
North America	USA	Concordia University Irvine
North America	USA	Concordia University Nebraska
North America	USA	Concordia University, St. Paul
North America	USA	Converse University
North America	USA	Coppin State University
North America	USA	Corban University
North America	USA	Cumberland University
North America	USA	Daemen University
North America	USA	Dakota State University
North America	USA	Dakota Wesleyan University
North America	USA	Dallas Baptist University
North America	USA	Dallas International University
North America	USA	Davenport University
North America	USA	Des Moines University
North America	USA	DeSales University
North America	USA	DeVry University
North America	USA	Dillard University
North America	USA	Divine Mercy University
North America	USA	Doane University
North America	USA	Dominican University (Illinois)
North America	USA	Dominican University New York
North America	USA	Dordt University
North America	USA	Drew University
North America	USA	D'Youville University
North America	USA	East Stroudsburg University of Pennsylvania
North America	USA	East Texas Baptist University
North America	USA	Eastern Connecticut State University
North America	USA	Eastern Mennonite University
North America	USA	Eastern University
North America	USA	East–West University
North America	USA	ECPI University
North America	USA	Edward Waters University
North America	USA	Elizabeth City State University
North America	USA	Elmhurst University
North America	USA	Elon University
North America	USA	Evangel University
North America	USA	Everest University
North America	USA	Everglades University

North America	USA	Fairfax University of America
North America	USA	Fairmont State University
North America	USA	Faulkner University
North America	USA	Fayetteville State University
North America	USA	Felician University
North America	USA	Ferris State University
North America	USA	Fielding Graduate University
North America	USA	Finlandia University
North America	USA	Fisk University
North America	USA	Florida A&M University
North America	USA	Florida Atlantic University
North America	USA	Florida Gulf Coast University
North America	USA	Florida Memorial University
North America	USA	Florida National University
North America	USA	Florida Polytechnic University
North America	USA	Fontbonne University
North America	USA	Fordham University
North America	USA	Framingham State University
North America	USA	Francis Marion University
North America	USA	Franciscan Missionaries of Our Lady University
North America	USA	Franciscan University of Steubenville
North America	USA	Franklin Pierce University
North America	USA	Franklin University
North America	USA	Freed–Hardeman University
North America	USA	Fresno Pacific University
North America	USA	Friends University
North America	USA	Full Sail University
North America	USA	Furman University
North America	USA	Future Generations University
North America	USA	Gallaudet University
North America	USA	Gannon University
North America	USA	Gardner–Webb University
North America	USA	George Fox University
North America	USA	Georgia Southwestern State University
North America	USA	Georgia Tech
North America	USA	Georgian Court University
North America	USA	Glenville State University
North America	USA	Golden Gate University
North America	USA	Goodwin University
North America	USA	Governors State University
North America	USA	Grace Christian University
North America	USA	Graceland University
North America	USA	Grambling State University
North America	USA	Grand Canyon University
North America	USA	Grand View University
North America	USA	Gwynedd Mercy University
North America	USA	Hallmark University
North America	USA	Harding University
North America	USA	Harrisburg University of Science and Technology
North America	USA	Harris–Stowe State University
North America	USA	Harvard University
North America	USA	Heidelberg University (Ohio)
North America	USA	Hellenic American University
North America	USA	Henderson State University
North America	USA	Heritage Christian University
North America	USA	Herzing University
North America	USA	High Point University
North America	USA	Hodges University
North America	USA	Hollins University
North America	USA	Holy Family University
North America	USA	Holy Names University
North America	USA	Hope International University
North America	USA	Houghton University
North America	USA	Houston Christian University
North America	USA	Howard Payne University
North America	USA	Humphreys University
North America	USA	Huntington University (United States)
North America	USA	Husson University
North America	USA	Huston–Tillotson University
North America	USA	Immaculata University
North America	USA	Indiana State University
North America	USA	Indiana University East
North America	USA	Indiana University Fort Wayne
North America	USA	Indiana University Kokomo
North America	USA	Indiana University South Bend

North America	USA	Indiana University Southeast
North America	USA	Indiana University–Purdue University Columbus
North America	USA	Indiana University–Purdue University Indianapolis
North America	USA	Indiana Wesleyan University
North America	USA	International Technological University
North America	USA	Iowa Wesleyan University
North America	USA	Jacksonville University
North America	USA	Jarvis Christian University
North America	USA	Jessup University
North America	USA	John Brown University
North America	USA	John Carroll University
North America	USA	John Paul the Great Catholic University
North America	USA	Johns Hopkins University
North America	USA	Johnson & Wales University
North America	USA	Johnson C. Smith University
North America	USA	Johnson University
North America	USA	Johnson University Florida
North America	USA	Judson University
North America	USA	Kansas Wesleyan University
North America	USA	Keiser University
North America	USA	Kennesaw State University
North America	USA	Kentucky Christian University
North America	USA	Kentucky State University
North America	USA	Kettering University
North America	USA	King University
North America	USA	La Roche University
North America	USA	La Salle University
North America	USA	La Sierra University
North America	USA	Lakeland University
North America	USA	Lamar University
North America	USA	Lander University
North America	USA	Langston University
North America	USA	Lasell University
North America	USA	Lawrence Technological University
North America	USA	Lawrence University
North America	USA	Lee University
North America	USA	Lehigh University
North America	USA	Lenoir–Rhyne University
North America	USA	Lesley University
North America	USA	LeTourneau University
North America	USA	Lewis University
North America	USA	Life Pacific University
North America	USA	Life University
North America	USA	Limestone University
North America	USA	Lincoln Christian University
North America	USA	Lincoln University
North America	USA	Lincoln University (California)
North America	USA	Lincoln University (Missouri)
North America	USA	Linfield University
North America	USA	Lipscomb University
North America	USA	Loma Linda University
North America	USA	Long Island University
North America	USA	Louisiana Christian University
North America	USA	Louisiana State University
North America	USA	Louisiana State University at Eunice
North America	USA	Louisiana State University Shreveport
North America	USA	Louisiana Tech University
North America	USA	Lourdes University
North America	USA	Loyola Marymount University
North America	USA	Loyola University New Orleans
North America	USA	Lubbock Christian University
North America	USA	Lynn University
North America	USA	Madonna University
North America	USA	Manchester University (Indiana)
North America	USA	Maranatha Baptist University
North America	USA	Marian University (Indiana)
North America	USA	Marian University (Wisconsin)
North America	USA	Marquette University
North America	USA	Martin University
North America	USA	Mary Baldwin University
North America	USA	Marymount University
North America	USA	Maryville University
North America	USA	Marywood University
North America	USA	Massachusetts Institute of Technology
North America	USA	McKendree University

North America	USA	McMurry University
North America	USA	McNeese State University
North America	USA	Mercer University
North America	USA	Mercyhurst University
North America	USA	Messiah University
North America	USA	Methodist University
North America	USA	Metropolitan State University
North America	USA	Metropolitan State University of Denver
North America	USA	Miami Regional University
North America	USA	Miami University
North America	USA	Mid-America Christian University
North America	USA	MidAmerica Nazarene University
North America	USA	Mid-Atlantic Christian University
North America	USA	Middle Georgia State University
North America	USA	Midland University
North America	USA	Midway University
North America	USA	Midwest University
North America	USA	Midwestern University
North America	USA	Milligan University
North America	USA	Millikin University
North America	USA	Minerva University
North America	USA	Minot State University
North America	USA	Misericordia University
North America	USA	Mississippi University for Women
North America	USA	Mississippi Valley State University
North America	USA	Missouri Baptist University
North America	USA	Missouri State University–West Plains
North America	USA	Molloy University
North America	USA	Monmouth University
North America	USA	Montana State University Billings
North America	USA	Montana State University–Northern
North America	USA	Montana Technological University
North America	USA	Moravian University
North America	USA	Morehead State University
North America	USA	Morgan State University
North America	USA	Mount Marty University
North America	USA	Mount Mary University
North America	USA	Mount Mercy University
North America	USA	Mount Saint Mary's University, Los Angeles
North America	USA	Mount St. Joseph University
North America	USA	Mount St. Mary's University
North America	USA	Mountain State University
North America	USA	Multnomah University
North America	USA	Muskingum University
North America	USA	Naropa University
North America	USA	National American University
North America	USA	National Louis University
North America	USA	National University (California)
North America	USA	Navajo Technical University
North America	USA	Nebraska Wesleyan University
North America	USA	Neumann University
North America	USA	New Jersey City University
North America	USA	New Jersey Institute of Technology
North America	USA	Newman University, Wichita
North America	USA	Niagara University
North America	USA	Norfolk State University
North America	USA	North American University
North America	USA	North Carolina A&T State University
North America	USA	North Carolina Central University
North America	USA	North Carolina Wesleyan University
North America	USA	North Central University
North America	USA	North Park University
North America	USA	Northeastern Illinois University
North America	USA	Northern Arizona University
North America	USA	Northern Illinois University
North America	USA	Northern Kentucky University
North America	USA	Northern State University
North America	USA	Northwest Nazarene University
North America	USA	Northwood University
North America	USA	Norwich University
North America	USA	Notre Dame de Namur University
North America	USA	Notre Dame of Maryland University
North America	USA	Nova Southeastern University
North America	USA	Oakland City University
North America	USA	Oakland University

North America	USA	Oakwood University
North America	USA	Oglethorpe University
North America	USA	Ohio Christian University
North America	USA	Ohio Dominican University
North America	USA	Oikos University
North America	USA	Okan International University
North America	USA	Oklahoma Baptist University
North America	USA	Oklahoma Christian University
North America	USA	Oklahoma City University
North America	USA	Oklahoma State University Institute of Technology
North America	USA	Oklahoma State University–Tulsa
North America	USA	Oklahoma Wesleyan University
North America	USA	Olivet Nazarene University
North America	USA	Open Christian University
North America	USA	Oral Roberts University
North America	USA	Oregon Health & Science University
North America	USA	Oregon Institute of Technology
North America	USA	Ottawa University
North America	USA	Ouachita Baptist University
North America	USA	Our Lady of the Lake University
North America	USA	Pace University
North America	USA	Palo Alto University
North America	USA	Park University
North America	USA	Patten University
North America	USA	Pennsylvania Institute of Technology
North America	USA	Pepperdine University
North America	USA	Pfeiffer University
North America	USA	Point Loma Nazarene University
North America	USA	Point Park University
North America	USA	Point University
North America	USA	Portland State University
North America	USA	Post University
North America	USA	Prairie View A&M University
North America	USA	Purdue University Global
North America	USA	Purdue University Northwest
North America	USA	Quincy University
North America	USA	Quinnipiac University
North America	USA	Randall University
North America	USA	Regent University
North America	USA	Regis University
North America	USA	Reinhardt University
North America	USA	Rider University
North America	USA	Robert Morris University
North America	USA	Rochester University
North America	USA	Roosevelt University
North America	USA	Rosalind Franklin University of Medicine and Science
North America	USA	Rowan University
North America	USA	Rush University
North America	USA	Sacred Heart University
North America	USA	Saginaw Valley State University
North America	USA	Saint Elizabeth University
North America	USA	Saint Joseph's University
North America	USA	Saint Leo University
North America	USA	Saint Peter's University
North America	USA	Saint Xavier University
North America	USA	Salem University
North America	USA	Sam Houston State University
North America	USA	Samford University
North America	USA	Samuel Merritt University
North America	USA	San Diego State University
North America	USA	San Diego University for Integrative Studies
North America	USA	Santa Clara University
North America	USA	Savannah State University
North America	USA	Saybrook University
North America	USA	Schiller International University
North America	USA	Schreiner University
North America	USA	Selma University
North America	USA	Seton Hall University
North America	USA	Seton Hill University
North America	USA	Shaw University
North America	USA	Shenandoah University
North America	USA	Shepherd University
North America	USA	Siena Heights University
North America	USA	Simmons University
North America	USA	Simpson University

North America	USA	Sinte Gleska University
North America	USA	Slippery Rock University
North America	USA	Soka University of America
North America	USA	South Carolina State University
North America	USA	South University
North America	USA	Southeastern Louisiana University
North America	USA	Southeastern University (Florida)
North America	USA	Southern Adventist University
North America	USA	Southern Arkansas University Tech
North America	USA	Southern Methodist University
North America	USA	Southern Nazarene University
North America	USA	Southern New Hampshire University
North America	USA	Southern Oregon University
North America	USA	Southern States University
North America	USA	Southern University
North America	USA	Southern University at New Orleans
North America	USA	Southern University at Shreveport
North America	USA	Southern Utah University
North America	USA	Southern Wesleyan University
North America	USA	Southwest Minnesota State University
North America	USA	Southwestern Assemblies of God University
North America	USA	Southwestern Christian University
North America	USA	Southwestern University
North America	USA	Spalding University
North America	USA	Spring Arbor University
North America	USA	St. Ambrose University
North America	USA	St. Andrews University (North Carolina)
North America	USA	St. Augustine's University (North Carolina)
North America	USA	St. Bonaventure University
North America	USA	St. Catherine University
North America	USA	St. John Fisher University
North America	USA	St. John's University (New York City)
North America	USA	St. Joseph's University (New York)
North America	USA	St. Mary's University, Texas
North America	USA	St. Thomas University (Florida)
North America	USA	State University of New York at Canton
North America	USA	State University of New York at Cortland
North America	USA	State University of New York at Delhi
North America	USA	State University of New York at Fredonia
North America	USA	State University of New York at Geneseo
North America	USA	State University of New York at Morrisville
North America	USA	State University of New York at Old Westbury
North America	USA	State University of New York at Oneonta
North America	USA	State University of New York at Potsdam
North America	USA	Stephen F. Austin State University
North America	USA	Stetson University
North America	USA	Stevens Institute of Technology
North America	USA	Stevenson University
North America	USA	Stratford University
North America	USA	Strayer University
North America	USA	Sul Ross State University
North America	USA	Sullivan University
North America	USA	Syracuse University
North America	USA	Taylor University
North America	USA	Temple University Ambler
North America	USA	Tennessee State University
North America	USA	Tennessee Wesleyan University
North America	USA	Texas A&M International University
North America	USA	Texas A&M University–Kingsville
North America	USA	Texas A&M University–Texarkana
North America	USA	Texas Christian University
North America	USA	Texas Lutheran University
North America	USA	Texas Southern University
North America	USA	Texas Wesleyan University
North America	USA	Texas Woman's University
North America	USA	Thomas Edison State University
North America	USA	Thomas Jefferson University
North America	USA	Thomas University
North America	USA	Tiffin University
North America	USA	Touro University
North America	USA	Touro University California
North America	USA	Touro University Nevada
North America	USA	Trevecca Nazarene University
North America	USA	Trident University International
North America	USA	Trine University

North America	USA	Trinity International University
North America	USA	Trinity Southwest University
North America	USA	Trinity Washington University
North America	USA	Troy University
North America	USA	Truett McConnell University
North America	USA	Truman State University
North America	USA	Tufts University
North America	USA	Tusculum University
North America	USA	Tuskegee University
North America	USA	Union Institute & University
North America	USA	University at Albany, SUNY
North America	USA	University at Buffalo
North America	USA	University of Advancing Technology
North America	USA	University of Akron
North America	USA	University of Alaska Anchorage
North America	USA	University of Alaska Southeast
North America	USA	University of Antelope Valley
North America	USA	University of Arkansas
North America	USA	University of Arkansas at Monticello
North America	USA	University of Arkansas at Pine Bluff
North America	USA	University of Arkansas Rich Mountain
North America	USA	University of Arkansas–Fort Smith
North America	USA	University of Baltimore
North America	USA	University of California, Berkeley
North America	USA	University of California, Riverside
North America	USA	University of California, San Francisco
North America	USA	University of California, Santa Cruz
North America	USA	University of Central Oklahoma
North America	USA	University of Charleston
North America	USA	University of Colorado Colorado Springs
North America	USA	University of Colorado Denver
North America	USA	University of Delaware
North America	USA	University of Detroit Mercy
North America	USA	University of Dubuque
North America	USA	University of Evansville
North America	USA	University of Findlay
North America	USA	University of Fort Lauderdale
North America	USA	University of Hartford
North America	USA	University of Hawaiʻi – West Oʻahu
North America	USA	University of Hawaiʻi at Mānoa
North America	USA	University of Holy Cross
North America	USA	University of Houston
North America	USA	University of Houston–Downtown
North America	USA	University of Houston–Victoria
North America	USA	University of Illinois Springfield
North America	USA	University of Illinois Urbana-Champaign
North America	USA	University of Indianapolis
North America	USA	University of Iowa
North America	USA	University of Jamestown
North America	USA	University of La Verne
North America	USA	University of Louisiana at Monroe
North America	USA	University of Louisville
North America	USA	University of Lynchburg
North America	USA	University of Maine at Augusta
North America	USA	University of Maine at Machias
North America	USA	University of Mary Hardin–Baylor
North America	USA	University of Maryland Global Campus
North America	USA	University of Maryland, Baltimore
North America	USA	University of Maryland, Baltimore County
North America	USA	University of Massachusetts Amherst
North America	USA	University of Massachusetts Boston
North America	USA	University of Massachusetts Global
North America	USA	University of Memphis
North America	USA	University of Michigan
North America	USA	University of Michigan–Dearborn
North America	USA	University of Minnesota Crookston
North America	USA	University of Minnesota Duluth
North America	USA	University of Minnesota Rochester
North America	USA	University of Missouri
North America	USA	University of Missouri–Kansas City
North America	USA	University of Missouri–St. Louis
North America	USA	University of Mobile
North America	USA	University of Montevallo
North America	USA	University of Mount Union
North America	USA	University of Nebraska Omaha

North America	USA	University of New Hampshire
North America	USA	University of New Haven
North America	USA	University of New Orleans
North America	USA	University of North Carolina at Asheville
North America	USA	University of North Carolina at Chapel Hill
North America	USA	University of North Carolina at Charlotte
North America	USA	University of North Georgia
North America	USA	University of North Texas
North America	USA	University of North Texas at Dallas
North America	USA	University of Northern Colorado
North America	USA	University of Northwestern Ohio
North America	USA	University of Oklahoma
North America	USA	University of Phoenix
North America	USA	University of Pikeville
North America	USA	University of Pittsburgh at Titusville
North America	USA	University of Portland
North America	USA	University of Redlands
North America	USA	University of Richmond
North America	USA	University of Rochester
North America	USA	University of Saint Joseph
North America	USA	University of Saint Mary
North America	USA	University of San Francisco
North America	USA	University of Science and Arts of Oklahoma
North America	USA	University of Scranton
North America	USA	University of Silicon Valley
North America	USA	University of Sioux Falls
North America	USA	University of South Alabama
North America	USA	University of South Carolina
North America	USA	University of South Carolina Beaufort
North America	USA	University of Southern Indiana
North America	USA	University of Southern Maine
North America	USA	University of St. Francis
North America	USA	University of St. Thomas (Minnesota)
North America	USA	University of St. Thomas (Texas)
North America	USA	University of Tennessee at Chattanooga
North America	USA	University of Tennessee Southern
North America	USA	University of Texas at Arlington
North America	USA	University of Texas at Dallas
North America	USA	University of Texas at Tyler
North America	USA	University of the Cumberlands
North America	USA	University of the District of Columbia
North America	USA	University of the Incarnate Word
North America	USA	University of the Pacific (United States)
North America	USA	University of the People
North America	USA	University of the Potomac
North America	USA	University of the Southwest
North America	USA	University of the West
North America	USA	University of Tulsa
North America	USA	University of Valley Forge
North America	USA	University of Virginia
North America	USA	University of Washington
North America	USA	University of West Florida
North America	USA	University of West Georgia
North America	USA	University of West Los Angeles
North America	USA	University of Western States
North America	USA	University of Wisconsin–La Crosse
North America	USA	University of Wisconsin–Stevens Point
North America	USA	University of Wisconsin–Stout
North America	USA	Upper Iowa University
North America	USA	Utah Tech University
North America	USA	Utah Valley University
North America	USA	Utica University
North America	USA	Valdosta State University
North America	USA	Valley City State University
North America	USA	Valparaiso University
North America	USA	Villanova University
North America	USA	Vincennes University
North America	USA	Virginia Commonwealth University
North America	USA	Virginia State University
North America	USA	Virginia Union University
North America	USA	Virginia University of Lynchburg
North America	USA	Viterbo University
North America	USA	Voorhees University
North America	USA	Wake Forest University
North America	USA	Walden University

North America	USA	Waldorf University
North America	USA	Walsh University
North America	USA	Warner Pacific University
North America	USA	Warner University
North America	USA	Washington Adventist University
North America	USA	Washington and Lee University
North America	USA	Wayland Baptist University
North America	USA	Wayne State University
North America	USA	Waynesburg University
North America	USA	Webber International University
North America	USA	Wesleyan University
North America	USA	West Virginia State University
North America	USA	West Virginia University at Parkersburg
North America	USA	West Virginia University Institute of Technology
North America	USA	Westcliff University
North America	USA	Western Carolina University
North America	USA	Western Colorado University
North America	USA	Western Governors University
North America	USA	Western Kentucky University
North America	USA	Western Michigan University
North America	USA	Western New England University
North America	USA	Western Oregon University
North America	USA	Westfield State University
North America	USA	Wheeling University
North America	USA	Widener University
North America	USA	Wilberforce University
North America	USA	Wilkes University
North America	USA	Willamette University
North America	USA	William Carey University
North America	USA	William Howard Taft University
North America	USA	William Peace University
North America	USA	William Penn University
North America	USA	William Woods University
North America	USA	Williams Baptist University
North America	USA	Wilmington University
North America	USA	Winston-Salem State University
North America	USA	Wittenberg University
North America	USA	Woodbury University
North America	USA	Worcester State University
North America	USA	Xavier University
North America	USA	Xavier University of Louisiana
North America	USA	Yeshiva University
North America	USA	Youngstown State University
Oceania	Australia	Central Queensland University
Oceania	Australia	Charles Darwin University
Oceania	Australia	Charles Sturt University
Oceania	Australia	Curtin University
Oceania	Australia	Deakin University
Oceania	Australia	Edith Cowan University
Oceania	Australia	Queensland University of Technology
Oceania	Australia	Royal Melbourne Institute of Technology
Oceania	Australia	Southern Cross University
Oceania	Australia	Swinburne University of Technology
Oceania	Australia	University of Canberra
Oceania	Australia	University of South Australia
Oceania	New Zealand	Auckland University of Technology
